# m^6^A Reader PRRC2A Promotes Colorectal Cancer Progression via CK1ε‐Mediated Activation of WNT and YAP Signaling Pathways

**DOI:** 10.1002/advs.202406935

**Published:** 2024-11-24

**Authors:** Xi Wu, Shiyang Wang, Yuwei Pan, Mengzhen Li, Manyu Song, Hanfu Zhang, Min Deng, Xu Yang, Jiuzhi Xu, Shuo Zhang, Jinhua Zhang, Fengchao Wang, Maksim V. Plikus, Cong Lv, Lu Yu, Zhengquan Yu

**Affiliations:** ^1^ The First Affiliated Hospital of Zhengzhou University Tianjian Laboratory of Advanced Biomedical Sciences Academy of Medical Sciences Zhengzhou University Zhengzhou Henan 450052 China; ^2^ State Key Laboratory of Animal Biotech Breeding College of Biological Sciences China Agricultural University Beijing 100193 China; ^3^ Key Laboratory of Precision Nutrition and Food Quality Ministry of Education Department of Nutrition and Health China Agricultural University Beijing 100193 China; ^4^ The college of Life Science and Bioengineering Beijing Jiaotong University Beijing 100044 China; ^5^ National Institute of Biological Science Beijing 102206 China; ^6^ Department of Developmental and Cell Biology Sue and Bill Gross Stem Cell Research Center Center for Complex Biological Systems University of California Irvine CA 92697 USA

**Keywords:** ATF1, CK1ε, colorectal cancer, Hippo/YAP pathway, PRRC2A, WNT pathway

## Abstract

Colorectal cancer (CRC) is the third most common cancer type and the second highest mortality rate among cancers. However, the mechanisms underlying CRC progression remain to be fully understood. In this work, a recently identified m^6^A‐modified RNA reader protein Proline‐rich Coiled‐coil 2a (PRRC2A) is markedly upregulated in CRC, and intestinal epithelium‐specific deletion of *Prrc2a* significantly suppressed tumor cell growth, stemness, and migratory capacity, while its overexpression promoted these behaviors. Through multiomics analysis, PRRC2A directly targeted *CSNK1E* (encoding CK1ε), maintaining its RNA stability in an m^6^A‐dependent manner, and that elevated CK1ε can concomitantly result in activation of the WNT and YAP signaling pathways. Interestingly, *PRRC2A* is directly regulated by the transcription factor ATF1 in its promoter. In summary, the work reveals a novel mechanism by which m^6^A reader PRRC2A promotes colorectal cancer progression via CK1ε and aberrant upregulation of WNT and YAP signaling. Therefore, PRRC2A and CK1ε can be potential therapeutic targets for treating CRC.

## Graphical abstract

m^6^A reader PRRC2A is aberrantly upregulated in colorectal cancer and associated with poor survival. Elevated PRRC2A promotes colorectal cancer progression by enhancing the stemness and migrative capacity of cancer cells. Mechanistically, ATF1‐mediated PRRC2A upregulation directly targets CSNK1E (encoding CK1ε) in an m^6^A‐dependent manner, which activates pro‐oncogenic WNT and YAP signaling pathways. This work provides potential therapeutic targets for colorectal cancer.

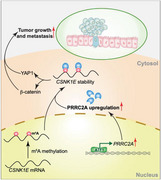

## 1 Introduction

Colorectal cancer (CRC) is a common digestive tract tumor with the third highest incidence rate and the second highest mortality rate among cancers, posing a serious threat to human life and health worldwide.^[^ ^1^, ^2^ ^]^ Understanding the mechanism underlying cancer progression is the foundation for the development of successful strategies for effective cancer prevention and management. N^6^‐methyladenosine (m^6^A) modification is the most prevalent internal modification in eukaryotic mRNAs, and its dysregulation plays a critical role in promoting cancer progression.^[^ ^3^, ^4^, ^5^ ^]^ m^6^A modifications of target RNAs need to be recognized by m^6^A reader proteins to regulate RNA export, splicing, translation, and degradation.^[^ ^3^, ^6^ ^]^ Several m^6^A reader proteins have been identified,^[^ ^3^ ^]^ including YTHDF1‐3, IGF2BP1‐3, and YTHDC1‐2. It has been reported that YTHDF2 stabilizes *MYC* mRNA to enhance glucose metabolism in glioblastoma stem cells.^[^ ^7^ ^]^ YTHDF1 also promotes CRC tumorigenesis and metastasis by increasing *ARHGEF2* translation.^[^ ^8^ ^]^ These reports indicate the importance of m^6^A reader proteins in contributing to cancer progression. PRRC2A is a newly identified m^6^A reader protein and it acts as a critical regulator of oligodendroglial specification and myelination.^[^ ^9^ ^]^ Interestingly, PRRC2A is upregulated in most cancers and is associated with poor survival.^[^ ^10^ ^]^ However, the functions of PRRC2A in CRC remain unknown.

As a key regulator of development, the WNT pathway is critical for intestinal stem cell maintenance and cancer progression.^[^ ^11^, ^12^, ^13^ ^]^ Canonical WNT/β‐catenin pathway is induced by extracellular WNT binding to its receptor complex Fzd and LRP5/6, resulting in inhibition of β‐catenin degradation by its destruction complex consisting of Axin, APC, and GSK3.^[^ ^13^, ^14^, ^15^ ^]^ Accumulated unphosphorylated β‐catenin then translocates into the nucleus to activate WNT target genes such as Cyclin D1 and Myc.^[^ ^16^, ^17^ ^]^ As a core component of the β‐catenin destruction complex, loss‐of‐function mutations of *APC* in germline cause familial adenomatous polyposis by activating the WNT signaling pathway, while somatic *APC* mutations are found in more than 80% of sporadic colorectal tumors.^[^ ^12^, ^18^, ^19^ ^]^ Therefore, activation of WNT signaling is a key step for CRC initiation.

Hippo/YAP signaling is another key driver for tissue growth in mammals. The core effectors YAP/TAZ translocate from the cytoplasm into the nucleus once the phosphorylation of YAP1 is removed.^[^ ^20^, ^21^ ^]^ Similar to the WNT pathway, YAP1 upregulation was noticed in over 80% of human CRC cases.^[^ ^22^ ^]^ In fact, although canonical WNT and YAP pathways seem to regulate distinct aspects of tissue development at different stages, emerging evidence has found them highly intertwined. It is believed that YAP/TAZ are components of the β‐catenin destruction complex, and their release from the complex is critical for activating WNT/β‐catenin signaling.^[^ ^23^ ^]^ A later study also demonstrated β‐catenin destruction complex‐independent regulation of YAP1 activation by APC.^[^ ^24^ ^]^ Most importantly, the gastrointestinal tract seems to be the most affected organ by WNT and YAP signaling, and crosstalk between them plays essential roles in crypt regeneration as well as in gut tumorigenesis.^[^ ^25^, ^26^ ^]^ However, coinciding activation mechanisms of WNT and YAP signaling remain to be uncovered.

Here we observed that *PRRC2A* expression was upregulated in CRC by transcription factor ATF1, and in turn, elevated PRRC2A directly increases the stability of *CSNK1E* mRNA (encoding CK1ε) in an m^6^A‐dependent manner, whereas CK1ε concomitantly activates both WNT and YAP signaling pathways, thus promoting CRC progression. This work identified m^6^A reader PRRC2A as an oncoprotein retaining cancer cell stemness and proliferation, revealing a unique mechanism for concurring WNT and YAP activation in CRC. More importantly, depletion of *PRRC2A* or *CSNK1E* suppressed the stemness of cancer cells and tumor growth, suggesting that this regulatory machinery could be of therapeutic interest for CRC.

## 2 Results

### 2.1 Elevated PRRC2A Expression Promotes Colon Tumor Progression

**Figure 1:**
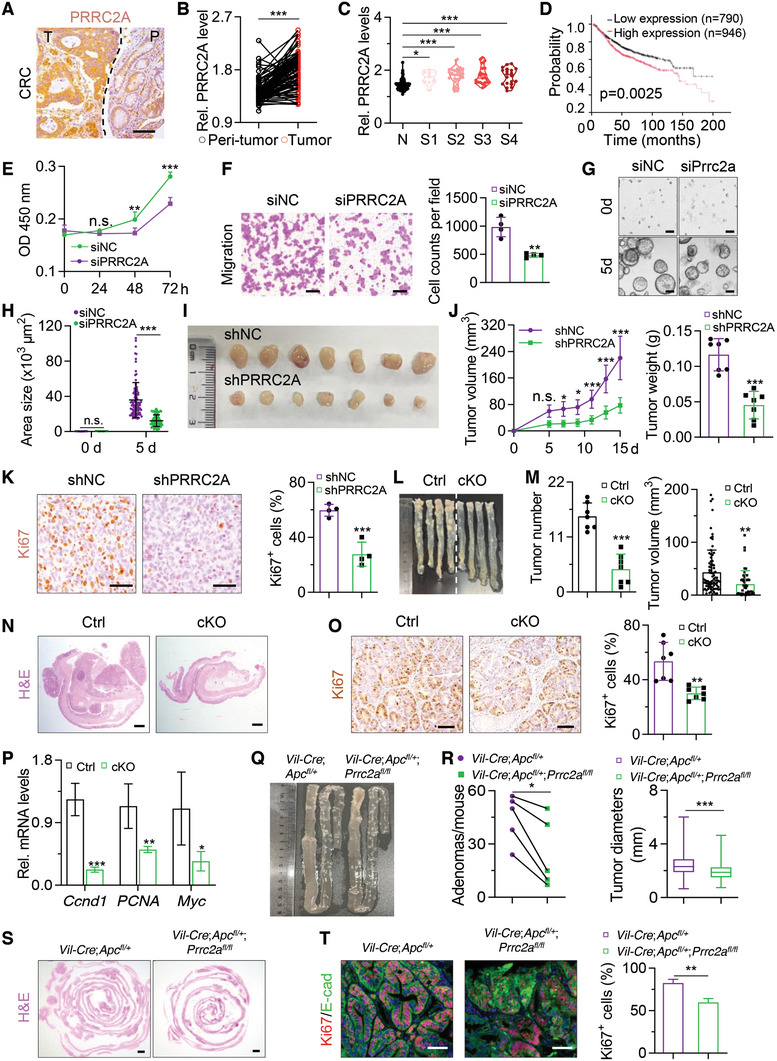
PRRC2A promotes CRC progression. A) Immunohistochemical staining of PRRC2A in human colon tumor tissues (T) and the corresponding peritumoral tissues (P). The dashed line indicates the border between colon tumor tissues and peritumoral tissues. Scale bar: 100 µm. B) Statistical analysis of PRRC2A expression based on immunohistochemical results in 80 paired colon adenocarcinoma/adjacent normal tissues. ***p < 0.001. C) Quantification of PRRC2A protein levels based on immunohistochemistry results in CRC patients with TNM stages 1 to 4. There were 80 peritumoral tissues and 9 CRC samples in stage 1, 28 in stage 2, 23 in stage 3, and 20 in stage 4. *p < 0.05, ***p < 0.001. D) High *PRRC2A* expression is associated with poor survival. *p *= 0.0025. E) Growth curve of HCT116 cells transfected with *PRRC2A* siRNA or siNC. *n* = 4. **p < 0.01, ***p < 0.001, n.s., *p* > 0.05. F) Representative images of transwell assay showing the migration of HCT116 cells transfected with siNC or siPRRC2A (left) and quantification of the migration of HCT116 cells (right). *n* = 4. **p < 0.01. Scale bar: 200 µm. G, H) Growth of APKS tumor organoids 5 days after *Prrc2a* siRNA transfection (G). Quantification of the organoid area as shown in panel H. *n* = 100 organoids at each time point. Scale bar: 100 µm. I) Images of xenograft tumors 15 days after transplantation with shNC‐ or shPRRC2A‐transfected HCT116 cells. *n* = 7. J) Tumor volume (left) and tumor weight (right) of xenografted tumors from HCT116 cells treated with shPRRC2A. *n* = 7. **p < 0.01, ***p < 0.001, n.s., *p* > 0.05. K) Immunohistochemical staining of Ki67 in the xenograft tumors in panel I (Left). The percentages of Ki67^+^ cells were quantified (right). *n* = 7. Scale bar: 50 µm. L, M) Representative gross images of AOM‐DSS colon tumors in control (Ctrl) and cKO mice (L). The numbers (left) and volume (right) of colon tumors were quantified (M). Ctrl: *n* = 84 tumors from 7 mice. cKO: *n* = 45 tumors from 7 mice. **p < 0.01, ***p < 0.001. N) Representative histological images of AOM‐DSS colon tumors in panel L. *n* = 7. Scale bar: 1 mm. O) Immunohistochemical staining of Ki67 of the colon tumors in panel L. The percentages of Ki67^+^ cells were quantified. *n* = 7. Scale bar: 50 µm. P) qRT‐PCR analysis of cell proliferation‐related genes *Ccnd1*, *PCNA*, and *Myc* in AOM‐DSS‐induced tumors harvested from Ctrl and cKO mice. *n* = 4. *p < 0.05, **p < 0.01, ***p < 0.001. Q,R) *Apc^fl/+^* mouse was crossed with *Vil‐cre* or cKO mice to induce intestinal tumors. Representative gross images of intestines harvested from 5‐month‐old *Vil‐cre*;*Apc^fl/+^* and *Vil‐cre*;*Apc^fl/+^*;*Prrc2a^fl/fl^* mice (Q). Tumor numbers and diameters were quantified (R). *Vil‐cre*;*Apc^fl/+^*: *n* = 223 tumors from 5 mice. *Vil‐cre*;*Apc^fl/+^*;*Prrc2a^fl/fl^*: *n* = 123 tumors from 5 mice. *p < 0.05, ***p < 0.001. S) Representative H&E staining images of intestinal tumors from 5‐month‐old *Vil‐cre*;*Apc^fl/+^* and *Vil‐cre*;*Apc^fl/+^*;*Prrc2a^fl/fl^* mice. *n* = 5. Scale bar: 1 mm. T) Immunofluorescence staining of Ki67 in tumors from 5‐month‐old *Vil‐cre*;*Apc^fl/+^* and *Vil‐cre*;*Apc^fl/+^*;*Prrc2a^fl/fl^* mice. Scale bar: 50 µm. The percentage of Ki67^+^ cells was quantified (right). *n* = 3. **p < 0.01. The data are presented as the means ± SDs. *p < 0.05; **p < 0.01; ***p < 0.001; n.s., *p* > 0.05. Statistical analysis in panels B and R was performed by paired Student's t‐test; One‐way ANOVA followed by Tukey's test was used in panels C; Two‐way ANOVA followed by Tukey's test was used in panels E and J; the rest was done by unpaired Student's t‐test.

Increasing evidence indicated that m^6^A modification plays a critical role in CRC progression.^[^ ^4^, ^8^, ^27^, ^28^, ^29^ ^]^ PRRC2A was recently identified as a novel m^6^A reader, and regulated oligodendroglial specification in brain development.^[^ ^9^ ^]^ To understand the roles of *PRRC2A* in CRC, we first analyzed transcriptome data from The Cancer Genome Atlas (TCGA) database and found the mRNA level of *PRRC2A* markedly elevated in human CRC tumors compared to normal tissues (Figure S1A, Supporting Information). We further verified this in human tissue sections and analyzed 80 pairs of colon adenocarcinoma/adjacent normal tissues from patients with distinct grades. Immunohistochemical results revealed consistent upregulation of PRRC2A protein in all stages of colorectal tumors (**Figure** **1**A–C). Consistently, upregulation of PRRC2A was also observed in colon tumors from the azoxymethane‐dextran sulfate sodium (AOM‐DSS) mouse model (Figure S1B, Supporting Information). We then analyzed the association between PRRC2A expression and well‐established gene mutations in CRC.^[^ ^30^ ^]^ Interestingly, we found that high expression levels of *PRRC2A* were significantly associated with *TP53* mutations in CRC patients (Figure S1C, Supporting Information). However, the expression of *PRRC2A* is not correlated to mutations of *KRAS*, *BRAF* (Figure S1D,E, Supporting Information). Consistently, western blotting analysis showed PRRC2A elevation in multiple human colorectal cancer cell lines (HCT116, HT29, LoVo, and SW480), compared to normal colon epithelial cell line NCM460 (Figure S1F, Supporting Information). Furthermore, we performed an Overall Survival (OS) analysis (Kaplan‐Meier analysis) using Kaplan‐Meier Plotter (http://www.kmplot.com), and found that high expression levels of *PRRC2A* were significantly correlated with poor survival in CRC patients (Figure 1D). Taken together, these findings showed that PRRC2A is upregulated in CRC tumors and associated with poor survival of CRC patients.

Next, we investigated the roles of PRRC2A in cell growth and migration by generating *PRRC2A*‐knockdown HCT116 cells and organoids (Figure S1G,H, Supporting Information). Knockdown of *PRRC2A* with siRNA abrogated proliferation and migration of colon cancer cells (Figure 1E,F; Figure S1I, Supporting Information). Moreover, in APKS mouse tumor organoids harboring mutations in *Apc*, *Tp53*, *Kras*, and *Smad4*, siRNA‐mediated *Prrc2a* knockdown significantly suppressed organoid growth, as evidenced by decreased organoid area after 5 days of culture (Figure 1G,H). Conversely, *PRRC2A* overexpression produced the opposite effects (Figure S1J–P, Supporting Information), suggesting that elevated PRRC2A was sufficient to promote cell proliferation and migration. Consistent with the in vitro data, the knockdown of *Prrc2a* with shRNA inhibited the growth of xenografted tumors in mice (Figure 1I‐K; Figure S1Q, Supporting Information). These results indicate that *PRRC2A* plays pro‐oncogenic roles in CRC.

To further elucidate the in vivo functions of *Prrc2a* during tumor development, we generated *Villin‐Cre*‐driven conditional *Prrc2a* knockout (cKO) mice (Figure S2A, Supporting Information), in which *Prrc2a* was specifically deleted in the intestinal epithelium (Figure S2B, Supporting Information).^[^ ^31^ ^]^ Under physiological conditions, *Prrc2a* is primarily expressed in the intestinal epithelium and in the lamina propria of the ileum and colorectum, while it is rare in the duodenum and jejunum (Figure S2C,D, Supporting Information). *Prrc2a* cKO mice were viable and fertile with no apparent gross phenotypes. No significant differences were observed in the colonic epithelium of cKO mice (Figure S2E–K, Supporting Information). These findings suggest that *Prrc2a* depletion may not affect intestinal homeostasis in physiology. To further elucidate its roles in cancerous conditions, we utilized the AOM‐DSS‐induced colorectal adenocarcinoma model, which recapitulates inflammation‐driven tumorigenesis. After AOM and DSS treatment, WT mice robustly developed multiple neoplasms in their colons, while *Prrc2a* deletion resulted in marked reductions in tumor burden, quantified by their size and number (Figure 1L–N). Expression of cell proliferation markers like Ki67, Cyclin D1, Myc, and PCNA also showed considerable declines as detected by immunostaining and qRT‐PCR analysis (Figure 1O,P). Apart from the carcinogen‐induced, colitis‐associated AOM‐DSS model, a mutant of *Apc* gene, a WNT pathway inhibitor, is another commonly used method to recapitulate familial CRC.^[^ ^32^ ^]^ Consistently, the pro‐oncogenic effects of *Prrc2a* were confirmed when *Prrc2a* was deleted in *Vil‐Cre*;*Apc^fl/+^* mice (Figure 1Q–T). Together, these findings demonstrated that Prrc2a was upregulated in CRC and promoted colon tumor progression.

### 2.2 PRRC2A Enhances Stemness of Colon Tumor Cells

**Figure 2:**
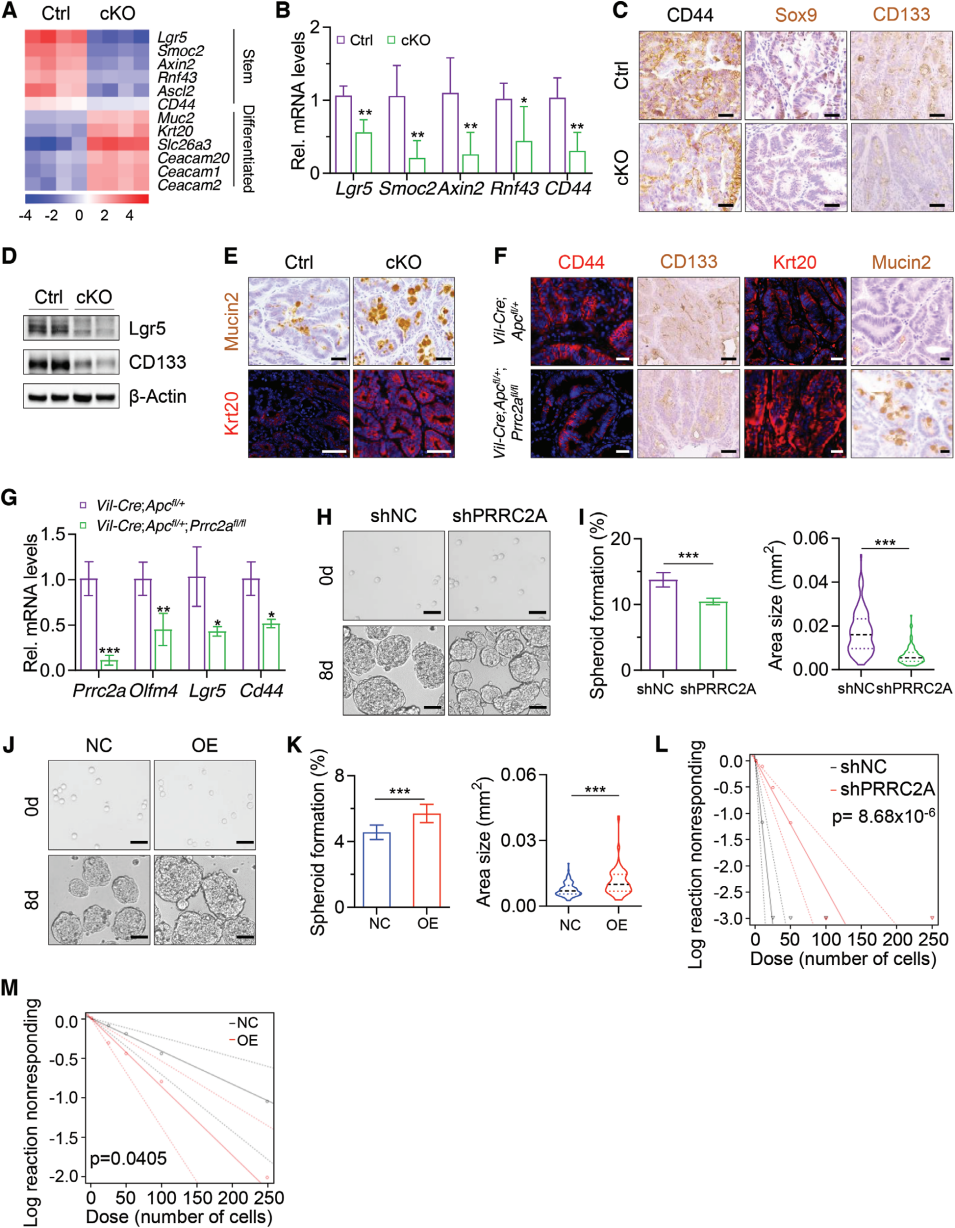
*Prrc2a* deficiency impaired stemness of colon tumor cells. A) Heatmap of differentially expressed genes (DEGs) associated with tumor cell stemness and differentiation in AOM‐DSS‐induced tumors from Ctrl and cKO mice. *n* = 4. B) qRT‐PCR analysis of cancer stem cell (CSC) marker genes *Lgr5*, *Smoc2*, *Axin2*, *Rnf43*, and *CD44* in AOM‐DSS‐induced tumors from Ctrl and cKO mice. *n* = 4. *p < 0.05, **p < 0.01. C) Immunohistochemical staining of CD44, Sox9, and Cd133 in colon tumors from Ctrl and cKO mice. n = 4. Scale bar: 50 µm. *n* = 4. Scale bar: 50 µm. D) Western blotting analysis of Lgr5 and CD133 in AOM‐DSS‐induced tumors from Ctrl and cKO mice. β‐Actin was used as a loading control. E) Immunostaining of Mucin2 and Krt20 in AOM‐DSS‐induced tumors from Ctrl and cKO mice. *n* = 4. Scale bar: 50 µm. F) Immunostaining of CD44, CD133, Krt20 and Mucin2 in intestinal tumors from 5‐month‐old *Vil‐cre*;*Apc^fl/+^* and *Vil‐cre*;*Apc^fl/+^*;*Prrc2a^fl/fl^* mice. *n* = 3. Scale bar: 25 µm. G) qRT‐PCR analysis of *Prrc2a*, *Olfm4*, *Lgr5* and *CD44* in intestinal tumors from 5‐month‐old *Vil‐cre*;*Apc^fl/+^* and *Vil‐cre*;*Apc^fl/+^*;*Prrc2a^fl/fl^* mice. n = 3. *p < 0.05, **p < 0.01, ***p < 0.001. H) Representative images of tumor spheroids formed by HCT116 cells. Cells were transfected with shPRRC2A or shNC plasmid, and images were taken at 0 days and 8 days after transfection. Scale bar: 200 µm. I) Quantification of the spheroid‐formation efficiency (see method) and area size from panel (H). For the spheroid‐formation efficiency, 3 biological replicates were included in each group; and 100 spheroids were counted for sphere area size. ***p < 0.001. J) Representative images of tumor spheroids formed by HCT116 cells after transfection with NC or PRRC2A overexpression plasmid at the indicated time points. *n* = 3. Scale bar: 200 µm. K) Quantification of spheroid‐formation efficiency and area in panel (J). For spheroid‐formation efficiency, 3 biological replicates were included in each group; and 100 spheroids were counted for sphere area size. ***p < 0.001. L, M) In vitro limiting dilution assay showing the spheroid formation frequency of diluted PRRC2A‐knockdown (L) or ‐overexpression (M) HCT116 cells. A well without spheres (diameter ≥ 50 µm) was defined as a non‐response (*n* = 10). The data are presented as the means ± SDs. *p < 0.05; **p < 0.01; ***p < 0.001; n.s., *p* > 0.05. Statistical analysis in panels B, G, I, K, and L was performed by unpaired Student's t‐test.

We next sought to investigate whether it can affect the stemness of colon cancer cells. Expression levels of cancer stem cell (CSC) marker genes *Lgr5*, *Smoc2*, *Axin2*, *Rnf43*, and *CD44* were markedly reduced in *Prrc2a*‐deficient tumors induced by AOM‐DSS treatment (**Figure** **2**A,B). Consistent with this finding, the abundances of tumor stem/progenitor cells, CD44^+^ cells, Sox9^+^ cells, and CD133^+^ cells, were markedly decreased in colon tumors from *Prrc2a*‐deficient mice (Figure 2C). Compared to the control group, the protein levels of CD133 and Lgr5 were significantly reduced in *Prrc2a*‐deficient intestinal tumors (Figure 2D). In contrast, the abundances of differentiated Mucin2^+^ cells and Krt20^+^ cells were elevated in cKO mice (Figure 2E). Similarly, loss of *Prrc2a* in *Vil‐Cre*;*Apc^fl/+^* mice also diminished the stemness of tumor cells and stimulated their differentiation (Figure 2F,G). To further investigate the direct effect of *Prrc2a* on cancer cell stemness, we induced CSC‐rich spheroids grown from a single HCT116 cell.^[^ ^33^ ^]^ Knockdown of *PRRC2A* inhibited the formation of HCT116 tumor spheroids, as quantified by the percentage of spheroids that had diameters larger than 75 µm (Figure 2H,I). Average area sizes of spheroids dramatically declined in the shPRRC2A‐transfected cells, while PRRC2A overexpression raised both spheroid‐forming frequency and area size (Figure 2J,K). Furthermore, in vitro limiting dilution assay^[^ ^34^ ^]^ showed that the knockdown of *PRRC2A* markedly suppressed tumor spheroid formation ability of HCT116 cells (Figure 2L). Conversely, *PRRC2A* overexpression promoted it (Figure 2M). These data indicate that PRRC2A is critical for maintaining the stemness of colon tumor cells.

In agreement with the importance of PRRC2A in maintaining tumor stemness, we found that *Prrc2a* deletion significantly repressed the organoid formation frequency and organoid growth of cultured intestinal and colonic crypts (Figure S3A–E, Supporting Information). The percentage of Ki67^+^ cells and Sox9^+^ cells markedly decreased in both intestinal and colon *Prrc2a*‐deficient organoids (Figure S3F,G, Supporting Information). In contrast, the percentage of differentiated Mucin2^+^ cells increased in organoids from cKO mice (Figure S3F,G, Supporting Information). These data indicated that Prrc2a is also essential for the stemness maintenance of intestinal epithelial cells.

### 2.3 Multiomics Analyses Identify PRRC2A Target Genes Involved in CRC

**Figure 3:**
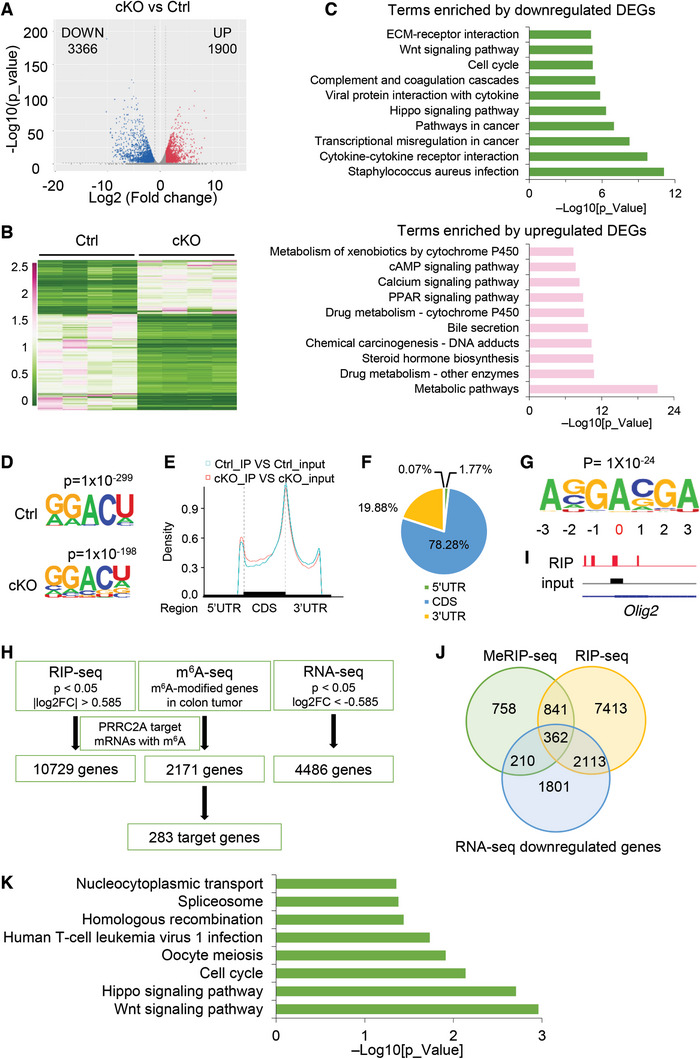
Multiomic analyses identify PRRC2A target genes involved in CRC. A, B) Volcano plot (A) and heatmap (B) showing the DEGs in AOM‐DSS‐induced tumors from Ctrl and cKO mice. *n* = 4. C) Top KEGG pathways enriched in the downregulated DEGs and upregulated DEGs in cKO tumors. D) m^6^A MeRIP‐seq was performed in Ctrl and cKO tumors. *n* = 4. The top sequence motifs enriched within m^6^A peaks were identified in Ctrl and cKO tumors. E) The density of m^6^A peaks in 3 nonoverlapping transcript segments (5′UTR, CDS, and 3′UTR). F) Pie chart showing the density of PRRC2A binding sites in HEK293T cells. RIP‐seq with an anti‐HA antibody was performed in HEK293T cells transfected with PRRC2A‐HA. G) A consensus PRRC2A binding motif was identified from the RIP‐seq data with the Hypergeometric Optimization of Motif EnRichment (HOMER) site. H) Schematic of the downstream analysis procedure based on RIP‐seq, MeRIP‐seq, and RNA‐seq data. I) Integrated Genomics Viewer (IGV) tracks the PRRC2A‐HA RIP (red) and input (black) signals in its known target gene *Olig2* mRNA. J) Venn diagram showing the overlap among downregulated DEGs identified by RNA‐seq, genes containing m^6^A peaks identified by MeRIP‐seq, and PRRC2A‐bound genes identified by PRRC2A‐HA RIP‐seq. K) KEGG pathway analysis of the 362 identified potential target genes of PRRC2A as shown in panel J. The data are presented as the means ± SDs. *p < 0.05; **p < 0.01; ***p < 0.001; n.s., *p* > 0.05.

We next sought to investigate the molecular mechanism underlying the pro‐oncogenic functions of Prrc2a. Considering that Prrc2a functions as a m^6^A reader protein, we performed m^6^A methylated RNA immunoprecipitation followed by sequencing (MeRIP‐seq; including input and immunoprecipitated samples) on tumor samples from Ctrl (*n* = 4) and cKO (*n* = 4) mice with AOM‐DSS‐induced colorectal adenoma. Analysis of sequencing data for the input samples revealed 3366 downregulated and 1900 upregulated genes (q_Value < 0.05) in cKO tumors compared to the Ctrl group (**Figure** **3**A,B). KEGG analysis revealed that downregulated genes were enriched in pathways such as “pathway in cancer”, “Hippo signaling pathway”, and “WNT signaling pathway”, while upregulated genes were enriched in “metabolic pathways” (Figure 3C). Consistent with previous reports,^[^ ^35^ ^]^ m^6^A MeRIP‐seq analysis showed that “GGAC” was the motif most enriched in m^6^A peaks in both Ctrl and cKO tumor cells (Figure 3D), thus indicating successful enrichment of m^6^A‐modified mRNA. We also observed consistent distribution of m^6^A peak enrichment near stop codons, while *Prrc2a* deletion had no effect on the overall m^6^A level (Figure 3E).

To identify PRRC2A target genes, we next performed PRRC2A‐HA RIP‐seq using an anti‐HA antibody to map RNA transcripts bound by PRRC2A (Figure S4A; Table S3, Supporting Information). PRRC2A binding sites were predominantly enriched in the coding regions and 3′UTRs of mRNA transcripts (Figure 3F), consistent with previous studies.^[^ ^9^ ^]^ Notably, the m^6^A‐modified “GGAC” motif showed high enrichment among PRRC2A binding motifs (Figure 3G), suggesting successful enrichment by this immunoprecipitation. PRRC2A‐HA RIP‐seq identified 10729 transcripts with greater than 1.5‐fold enrichment compared with the input RNA (Figure 3H). *Olig2*, a known target gene of PRRC2A,^[^ ^9^ ^]^ was identified among the enriched transcripts (Figure 3I), validating the RIP‐seq data. Gene Ontology (GO) analysis of the RIP‐seq data showed that PRRC2A‐bound RNAs were primarily associated with the terms “P53 pathway”, “cell cycle” and “RNA metabolic processes”, which are essential for cell proliferation and cancer progression (Figure S4B, Supporting Information). To identify potential PRRC2A target genes, we performed overlap analysis on RIP‐seq, m^6^A MeRIP‐seq, and RNA‐seq datasets, and identified a total of 362 overlapping potential PRRC2A target genes with m^6^A modifications (Figure 3I,J; Table S4, Supporting Information). KEGG pathway enrichment analysis showed that the enriched pathways included “WNT pathway” and “Hippo pathway” (Figure 3K), which are critical for the stemness of cancer cells.

### 2.4 PRRC2A Activates WNT and YAP Signaling Pathways in CRC

**Figure 4:**
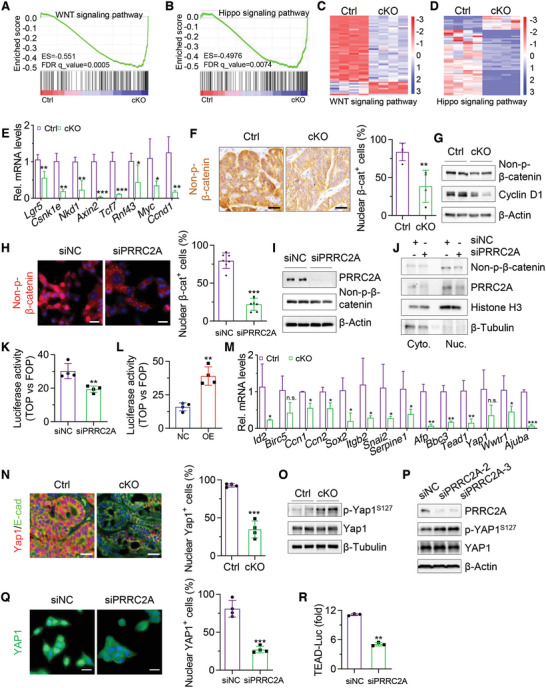
Elevated PRRC2A activates the WNT and YAP signaling pathways. A, B) Gene set enrichment assay (GSEA) showing WNT (A) and Hippo (B) signaling pathway genes are enriched in transcriptome profiles of AOM‐DSS‐induced tumors from Ctrl and cKO mice. *n* = 4. C, D) Heatmap showing DEGs associated with the WNT (C) and Hippo (D) signaling pathways in AOM‐DSS‐induced tumors from Ctrl and cKO mice. *n* = 4. E) qRT‐PCR analysis of WNT‐related genes in AOM‐DSS‐induced tumors from Ctrl and cKO mice. *n* = 3. *p < 0.05, **p < 0.01, ***p < 0.001. F) Representative immunohistochemical images of non‐p‐β‐catenin in AOM‐DSS‐induced colon tumors from Ctrl and cKO mice (left). Scale bar: 100 µm. The percentage of nuclear β‐catenin^+^ cells was quantified (right). *n* = 4. **p < 0.01. G) Western blotting analysis of non‐p‐β‐catenin and Cyclin D1 in AOM‐DSS‐induced tumors from Ctrl and cKO mice. β‐Actin was used as a loading control. H) Representative immunofluorescence images of non‐p‐β‐catenin in HCT116 cells transfected with siPRRC2A or siNC (left). Scale bar: 20 µm. The percentage of nuclear β‐catenin^+^ cells was quantified (right). *n* = 7. ***p < 0.001. I) Western blotting analysis of non‐p‐β‐catenin and PRRC2A in HCT116 cells transfected with siPRRC2A or siNC. β‐Actin was used as a loading control. J) Western blotting for non‐p‐β‐catenin in nuclear and cytoplasmic proteins isolated from HCT116 cells transfected with siNC or siPRRC2A. Histone H3 and β‐Tubulin were used as positive controls for nuclear and cytoplasmic proteins, respectively. K, L) Luciferase activity of the TOPflash reporter compared with that of the FOPflash reporter in HCT116 cells after transfection with siPRRC2A (K) or the PRRC2A overexpression vector (L). *n* = 4. **p < 0.01. M) qRT‐PCR analysis of Hippo‐related genes in AOM‐DSS‐induced tumors from Ctrl and cKO mice. *n* = 3. *p < 0.05, **p < 0.01, ***p < 0.001. n.s., *p* >0.05. N) Immunofluorescence staining of Yap1 in colon tumors from Ctrl and cKO mice. DAPI staining is shown in blue. Scale bar: 20 µm. The percentage of nuclear Yap1^+^ cells was quantified. *n* = 4. ***p < 0.001. O) Western blotting analysis of p‐YAP1 and YAP1 in AOM‐DSS‐induced tumors from Ctrl and cKO mice. β‐Tubulin was used as a loading control. P) Western blotting analysis of p‐YAP1, YAP1 and PRRC2A in HCT116 cells transfected with different siRNAs to knockdown PRRC2A. β‐Actin was used as a loading control. Q) Representative immunofluorescence images of YAP1 in HCT116 cells infected with siNC or siPRRC2A (left). DAPI staining is shown in blue. Scale bar: 20 µm. The percentage of nuclear YAP1^+^ cells was quantified (right). *n* = 4. ***p < 0.001. R) TEAD luciferase reporter assay showing that knockdown of PRRC2A with siRNA inhibits YAP1 activity. HCT116 cells were transfected with the indicated siRNAs and the YAP1 construct for 36 h before harvesting for the luciferase assay. *n* = 3. ***p < 0.001. The data are presented as the means ± SDs. *p < 0.05; **p < 0.01; ***p < 0.001; n.s., *p* > 0.05. Statistical analysis in panels E, F, H, K, L, N, Q, and R was performed by unpaired Student's t‐test.

The above results suggest that the activity of WNT and Hippo/YAP signaling pathways might be regulated by Prrc2a. To test this, Gene Set Enrichment Analysis (GSEA) on RNA‐seq data revealed that WNT and Hippo signaling pathways are significantly enriched in cKO tumors (**Figure** **4**A,B). Heatmap assay also showed that a list of WNT‐related and Hippo‐related genes are altered in cKO tumors (Figure 4C,D). The downregulation of WNT‐responsive genes, including *Lgr5*, *Csnk1e*, *Nkd1*, *Axin2*, *Rnf43*, *Sox9*, *Ccnd1* and *Myc* were validated by qRT‐PCR (Figure 4E). As the marker for activation of WNT pathway, the number of non‐phosphorylated, nuclear β‐catenin^+^ cells were markedly reduced in cKO mouse tumors (Figure 4F), and the protein levels of active β‐catenin and its target Cyclin D1 were markedly decreased (Figure 4G). Consistent with these findings, the knockdown of *Prrc2a* in HCT116 cells led to a reduction in nuclear β‐catenin^+^ cells (Figure 4H) and in the protein level of active β‐catenin (Figure 4I,J). Another commonly‐used method for WNT transcriptional activity detection is the TOPflash/FOPflash reporter assay which utilizes WNT activity‐sensitive or mutant, nonfunctional Tcf sites. As a result, the knockdown of *PRRC2A* suppressed WNT signaling activity, measured by the expression ratio between TOP and FOP (Figure 4K). Conversely, *PRRC2A* overexpression led to increases in nuclear β‐catenin^+^ cells (Figure S5A,B, Supporting Information) and in TOPflash/FOPflash reporter activity (Figure 4L). Furthermore, analysis of TCGA data showed positive correlations between the expression level of *PRRC2A* and WNT target gene *CCND1*, and the latter is also positively associated with poor survival in CRC patients (Figure S5C,D, Supporting Information). Taken together, these findings demonstrate that PRRC2A can activate WNT signaling pathway.

YAP1 is another major signal molecule for regulating organ growth and cancer progression. By analyzing multi‐omics sequencing data, we noticed that YAP pathway was also profoundly affected in the cKO tumors. qRT‐PCR analysis confirmed that many downstream target genes of YAP1, including *Ccn1* and *Ccn2*, were significantly downregulated in cKO mouse tumors (Figure 4M). Consistently, *Prrc2a* deficiency led to a marked reduction in nuclear YAP1^+^ cells (Figure 4N) and an increase in the levels of phosphorylated YAP1 (Yap1 activity is evaluated by the ratio of YAP1 to p‐YAP1) (Figure 4O). In agreement with the in vivo findings, the knockdown of *PRRC2A* in HCT116 cells resulted in increased YAP1 phosphorylation and decreased nuclear YAP1^+^ cells (Figure 4P,Q), while *PRRC2A* overexpression resulted in the opposite effects (Figure S5E,F, Supporting Information). Furthermore, luciferase reporter assay revealed that knockdown of *PRRC2A* in HCT116 cells significantly suppressed YAP1‐induced TEAD4 transactivation (Figure 4R), whereas overexpression of *PRRC2A* promoted it (Figure S5G, Supporting Information). Moreover, the regulatory effects of PRRC2A on YAP1 activity were confirmed in APKS organoids (Figure S5H–J, Supporting Information).^[^ ^36^ ^]^ Furthermore, analysis of human CRC tissues from TCGA data showed positive correlations between *PRRC2A* and *YAP1*, as well as between *PRRC2A* and YAP1's transcriptional coactivators *TEAD1* and *TEAD3* (Figure S5K–M, Supporting Information). Importantly, these genes are positively correlated with poor survival in CRC (Figure S5N–P, Supporting Information). Collectively, these findings demonstrate that PRRC2A activates WNT and YAP signaling pathways.

### 2.5 PRRC2A Directly Targets *CSNK1E* in an m^6^A‐dependent Manner that Enhances both WNT and YAP Activities

**Figure 5:**
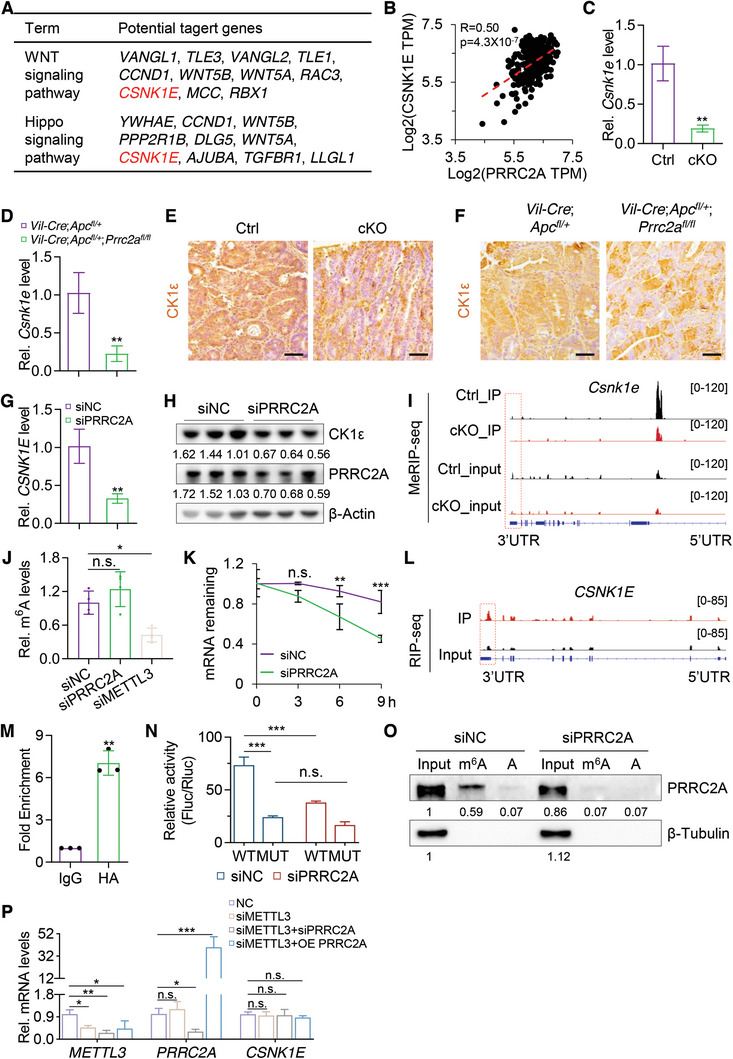
PRRC2A regulates CSNK1E expression in an m^6^A‐dependent manner. A) List of potential PRRC2A target genes involved in WNT and Hippo signaling pathways. B) Spearman correlation analysis of *CSNK1E* and *PRRC2A* (*p* < 0.001; R = 0.50) in human CRC based on TCGA database. C, D) qRT‐PCR for *Csnk1e* in AOM‐DSS tumors from Ctrl and cKO mice (C), and in tumors from 5‐month‐old *Vil‐cre*;*Apc^fl/+^* and *Vil‐cre*;*Apc^fl/+^*;*Prrc2a^fl/fl^* mice (D). *n* = 3 for each group. **p < 0.01. E, F) Representative immunohistochemical staining images of CK1ε in AOM‐DSS tumors from Ctrl and cKO mice (E), and in tumors from 5‐month‐old *Vil‐cre*;*Apc^fl/+^* and *Vil‐cre*;*Apc^fl/+^*;*Prrc2a^fl/fl^* mice (F). Scale bar: 50 µm. G) qRT‐PCR analysis of *CSNK1E* in LoVo cells transfected with siNC and siPRRC2A. **p < 0.01. H) Western blotting analysis of CK1ε and PRRC2A in LoVo cells after *PRRC2A* knockdown. β‐Actin was used as the loading control. I) m^6^A MeRIP IP and input signals located near the 3′UTR of *Csnk1e* mRNA (dotted box) in AOM‐DSS‐induced tumors from Ctrl and cKO mice. J) MeRIP‐qPCR analysis was performed to examine the level of m^6^A in *CSNK1E* 3′UTR in HCT116 cells with or without knockdown of PRRC2A or METTL3. *n* = 4. *p < 0.05; n.s., *p* > 0.05. K) LoVo cells were transfected with siNC or siPRRC2A and treated with actinomycin D, remained *CSNK1E* mRNA was measured at the indicated time points. The amount of residual RNA was normalized to the amount at 0 h. *n* = 3. **p < 0.01; ***p < 0.001; n.s., *p* > 0.05. L) HA RIP (red) and input (black) signals located near the 3′UTR of *CSNK1E* mRNA (dotted box). M) PRRC2A‐HA enrichment of *CSNK1E* mRNA in LoVo cells by RIP‐qPCR. Enrichment values are presented relative to those with IgG. *n* = 3. **p < 0.01. N) Luciferase activity in lysates of HCT116 cells transfected with a luciferase reporter vector containing the WT or mutated (MUT) *CSNK1E* 3′UTR sequence. Luciferase activities were measured in HCT116 cells with or without PRRC2A‐knockdown. *n* = 4. ***p < 0.001; n.s., *p* > 0.05. O) Western blotting analysis of PRRC2A in HCT116 cells after RNA pull‐down using single‐stranded CSNK1E RNA with (m^6^A) or without (A) m^6^A modifications. β‐Tubulin was used as a loading control. P) qRT‐PCR analysis of *METTL3*, *PRRC2A*, and *CSNK1E* mRNA expression in HCT116 cells transfected with siNC, siMETTL3, siMETTL3 with siPRRC2A, or siMETTL3 with PRRC2A overexpression vector. *n* = 3. *p < 0.05; **p < 0.01; ***p < 0.001; n.s., *p* > 0.05. The data are presented as the means ± SDs. *p < 0.05; **p < 0.01; ***p < 0.001; n.s., *p* > 0.05. Statistical analysis in panel J and P was performed by One‐way ANOVA followed by Tukey's test; Two‐way ANOVA followed by Tukey's test was used in panels K and N; the rest was done by unpaired Student's t‐test.

To explore the direct interactions between PRRC2A and its targeted transcripts that could regulate WNT and Hippo signaling pathways, we further analyzed the potential PRRC2A target genes associated with the 2 pathways. Among these genes, *CSNK1E* was selected for further validation (**Figure** **5**A), as it has been reported that CK1ε (encoded by *CSNK1E*) is a serine/threonine protein kinase that can activate WNT signaling^[^ ^37^ ^]^ and concomitantly modulate YAP1 activity.^[^ ^38^ ^]^ Analysis of TCGA data showed that *CSNK1E* is markedly upregulated in CRC at different stages (Figure S6A, Supporting Information). Consistent with the previous report, the knockdown of *CSNK1E* decreased, while *CSNK1E* overexpression increased, the protein level of active β‐catenin, supporting the idea that *CSNK1E* can activate WNT signaling in CRC (Figure S6B–E, Supporting Information). Similarly, the knockdown of *CSNK1E* suppressed YAP1 activity, while its overexpression activated it (Figure S6B–E, Supporting Information). Collectively, these findings demonstrate that CK1ε can activate both WNT and YAP signaling pathways in CRC, mediating the regulatory effect of PRRC2A on the 2 pathways.

To further investigate the regulatory relationship between *PRRC2A* and *CSNK1E*, we analyzed their correlation in UALCAN database, revealing a positive correlation between *CSNK1E* and *PRRC2A* expression (Figure 5B). In agreement, *Prrc2a* deletion led to the reduction in the RNA and protein levels of CK1ε in AOM‐DSS‐induced colon tumors and *Apc* mutation‐driven colorectal tumors (Figure 5C–F). In line with the in vivo findings, the knockdown of *PRRC2A* suppressed *CSNK1E* expression in CRC cells, while its overexpression upregulated it (Figure 5G,H; Figure S6F, Supporting Information). These findings indicate that PRRC2A positively regulates *CSNK1E* expression.

Next, we sought to investigate whether PRRC2A regulates *CSNK1E* expression in a m^6^A‐dependent manner. To this end, m^6^A site prediction utility SRAMP (http://www.cuilab.cn/sramp) was used, and only one m^6^A site in the 3′UTR of *CSNK1E* was identified (Figure S6G, Supporting Information). Consistent with this finding, MeRIP‐seq also identified one m^6^A site in 3′UTR of *CSNK1E* (Figure 5I). Given that *CSNK1E* 3′UTR is modified by m^6^A methylation, we then designed experiments to dissect the regulatory machinery of PRRC2A on *CSNK1E*. By performing MeRIP‐qPCR analysis with HCT116 cells, we found that the knockdown of *PRRC2A* did not affect the level of m^6^A of *CSNK1E* transcripts. In comparison, depletion of *METTL3*, the key enzyme of RNA methyltransferase complex,^[^ ^39^, ^40^ ^]^ significantly abolished *CSNK1E* 3′UTR m^6^A methylation (Figure 5J). It has been reported that PRRC2A functions as m^6^A reader that stabilizes target mRNAs.^[^ ^9^ ^]^ In agreement with this idea, the shortening of *CSNK1E* mRNA half‐life was observed upon *PRRC2A* inhibition (Figure 5K). Together, these results indicated that m^6^A reader PRRC2A regulates the expression of *CSNK1E* by stabilizing its mRNA. Next, we examined whether PRRC2A‐binding m^6^A sites exist on *CSNK1E* mRNA. PRRC2A RIP‐seq peaks were found to be enriched predominantly in 3′UTR of *CSNK1E* mRNA (Figure 5L). To validate this finding, we performed RIP‐qPCR analysis using anti‐HA‐tag antibody and observed enrichment of *CSNK1E* in the PRRC2A immunoprecipitate compared with IgG (Figure 5M), suggesting a direct interaction between PRRC2A and *CSNK1E* mRNA. Furthermore, we investigated whether the regulation of *CSNK1E* by PRRC2A is dependent on the m^6^A site in *CSNK1E* 3′UTR using dual luciferase assay. Knockdown of *PRRC2A* significantly reduced luciferase activity in the wild‐type (WT) group (*CSNK1E* gene WT m^6^A sites in the 3′UTR) but not in the mutant group (m^6^A sites mutated from A to T) (Figure 5N). In contrast, *PRRC2A* overexpression caused a substantial increase in luciferase activity (Figure S6H, Supporting Information). Moreover, RNA pull‐down assay demonstrated that m^6^A modification of *CSNK1E* RNA oligos greatly facilitated binding of PRRC2A to m^6^A‐modified site compared with that in the unmethylated *CSNK1E*‐A oligos, and this increase in the binding was markedly reduced upon *PRRC2A* suppression (Figure 5O). Interestingly, when *METTL3* was knocked down to reduce global m^6^A levels in HCT116 cells, *CSNK1E* mRNA levels were unaffected by either overexpression or inhibition of PRRC2A, demonstrating that PRRC2A's regulatory effects on *CSNK1E* expression depended on m^6^A modification (Figure 5P). Taken together, these findings indicate that PRRC2A increases the stability of *CSNK1E* mRNA by binding to m^6^A‐modified sites in its 3′UTR.

### 2.6 *CSNK1E* Overexpression Rescues the Inhibitory Effects of *PRRC2A* Knockdown on CRC Progression

**Figure 6:**
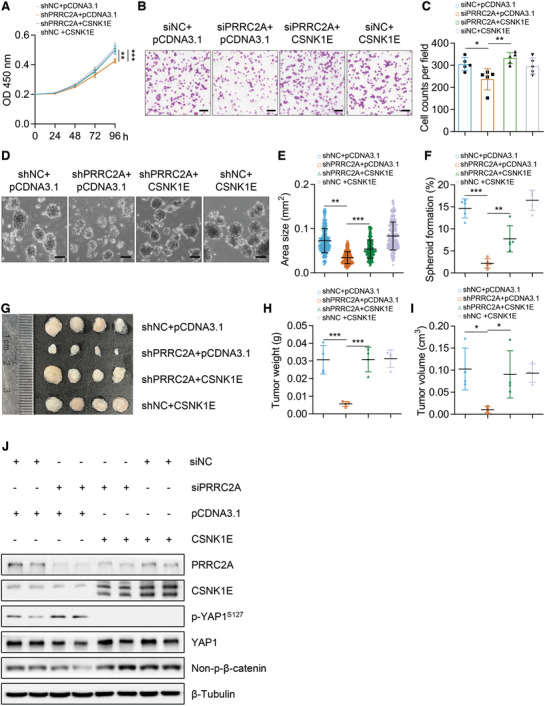
CSNK1E abolishes the inhibitory effects of *PRRC2A* suppression on tumor growth and stemness. A) Growth curve of shPRRC2A HCT116 cells with or without *CSNK1E* overexpression. siNC was used as the control. *n* = 5. ***p < 0.001. B, C) Transwell assay showing migration of HCT116 cells transfected with PRRC2A siRNAs and/or the *CSNK1E* overexpression vector (B). Quantification of cell migration was shown in panel (C) *n* = 5. **p < 0.01; **p < 0.01. Scale bar: 100 µm. D) Representative images of tumor spheroids at indicated conditions. HCT116 cells were transfected with shNC, shPRRC2A, or shPRRC2A + *CSNK1E* overexpressing plasmid, and then culture for 11 days. Scale bar: 100 µm. E, F) Area size (E) and spheroid‐formation efficiency (F) were quantified based on data in panel D. For the area size, 50 spheroid areas were counted for each group. For quantification of spheroid‐formation efficiency, 5 biological replicates were included in each group. **p < 0.01; ***p < 0.001. G) Image of xenograft tumors derived from HCT116 cells transfected with shNC, shPRRC2A, and shPRRC2A + *CSNK1E* overexpression vector. Tumors were harvested 2 weeks after transplantation. H, I) Statistical analysis of weights (H) and volumes (I) of xenograft tumors in panel G. *n* = 4 for each group. *p < 0.05; ***p < 0.001. J) Western blotting analysis for PRRC2A, CK1ε, p‐YAP1^S127^, YAP1, and non‐p‐β‐catenin in HCT116 cells transfected with NC, PRRC2A siRNAs and PRRC2A siRNA + *CSNK1E* overexpressing vector. The data are presented as the means ± SDs. *p < 0.05; **p < 0.01; ***p < 0.001; n.s., *p* > 0.05. Statistical analysis in panel C, E, F, H and I was performed by One‐way ANOVA followed by Tukey's test; the rest was done by unpaired Student's t‐test.

To further determine whether CK1ε functions as a critical downstream effector of PRRC2A in the context of CRC progression, we overexpressed *CSNK1E* in *PRRC2A*‐knockdown colon cancer cells and found that knockdown of *PRRC2A* suppressed cell proliferation and migration, while *CSNK1E* overexpression rescued the suppressive effects (**Figure** **6**A–C). Moreover, the knockdown of *PRRC2A* inhibited both spheroid‐forming efficiency and spheroid growth of CSCs, while *CSNK1E* overexpression restored the inhibitory effects on spheroid‐forming ability, suggesting that CK1ε mediates the effects of PRRC2A in maintaining proliferative capacity and stemness of CSCs (Figure 6D–F). Furthermore, tumor xenograft assay also demonstrated that *CSNK1E* overexpression reversed the inhibitory effect of *PRRC2A* knockdown on tumor growth (Figure 6G–I). Consistent with the reversal of these phenotypes, *CSNK1E* overexpression also reversed *PRRC2A* inhibition‐induced reductions in the activity of WNT and YAP pathways (Figure 6J). Taken together, our findings demonstrate that *CSNK1E* is a functional target gene of PRRC2A in CRC and mediates the pro‐oncogenic functions of PRRC2A.

### 2.7 Transcription Factor ATF1 Directly Upregulates *PRRC2A* in CRC Cells

**Figure 7:**
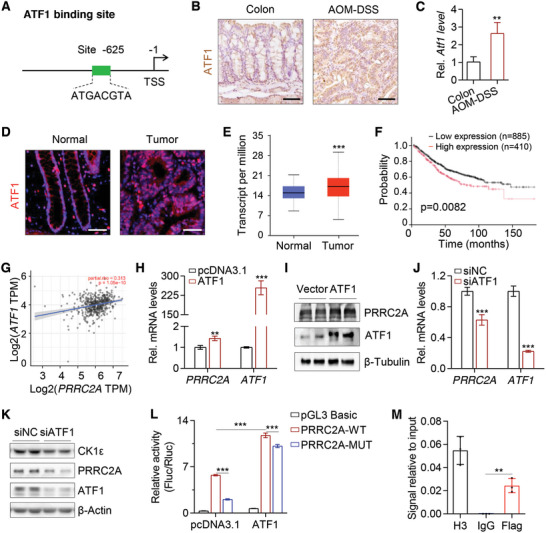
ATF1 transcriptionally upregulates PRRC2A in CRC cells. A) The schematic diagram showing a potential ATF1 binding site in the *PRRC2A* promoter. B) Immunohistochemical staining of ATF1 in normal mouse colon tissues and the AOM‐DSS‐induced mouse colon tumors. Scale bar: 50 µm. C) qRT‐PCR analysis of *Atf1* in normal mouse colon tissues and AOM‐DSS‐induced mouse colon tumors. *n* = 3. **p < 0.01. D) Immunofluorescent staining of ATF1 in human colorectal peritumor and tumor tissues from CRC patients. Scale bar: 50 µm. E) Box plots of *ATF1* expression in normal colorectal tissues and colorectal tumor tissues based on TCGA data. A total of 41 normal tissue samples and 286 CRC samples were included. ***p < 0.001. F) High *ATF1* expression is associated with poor survival of CRC patients. *p* = 0.0082. G) Spearman correlation analysis of *ATF1* and *PRRC2A* (*p* < 0.001; R = 0.313) in human CRC based on TCGA database. H) qRT‐PCR analysis of *PRRC2A and ATF1* in HCT116 cells transfected with pcDNA3.1 and *ATF1* overexpression vector. **p < 0.01; ***p < 0.001. I) Western blotting analysis of ATF1 and PRRC2A in HCT116 cells transfected with pcDNA3.1 and *ATF1* overexpression vector. β‐Tubulin was used as the loading control. J) qRT‐PCR analysis of *PRRC2A and ATF1* in HCT116 cells transfected with siNC or siATF1. ***p < 0.001. K) Western blotting analysis of CK1ε, ATF1, and PRRC2A in HCT116 cells transfected with siNC or siATF1. β‐Actin was used as the loading control. L) Luciferase activity in lysates of HCT116 cells transfected with a luciferase reporter vector containing the WT or mutated (MUT) *PRRC2A* promoter sequence. Luciferase activities were measured in HCT116 cells with or without *ATF1*‐overexpression. ***p < 0.001. M) ChIP‐qPCR analysis was performed to validate ATF1 enrichment in *PRRC2A* genomic DNA using HCT116 cells. Briefly, ChIP‐qPCR assay was carried out using an antibody against Flag on HCT116 cells transfected with an ATF1‐Flag overexpression vector. The antibody against Histone H3 was used as a positive control. IgG was used as a negative control. The enrichment of ATF1 binding to *PRRC2A* promoter was quantified using qPCR; *n* = 3 technical replicates. **p < 0.01. The data are presented as the means ± SDs. *p < 0.05; **p < 0.01; ***p < 0.001; n.s., *p* > 0.05. Statistical analysis in panel L was performed by Two‐way ANOVA followed by Tukey's test; the rest was done by unpaired Student's t‐test.

We have demonstrated that PRRC2A promoted CRC progression via WNT and YAP signaling pathways, however, the upstream mechanisms for PRRC2A upregulation in the context of CRC still remain unknown. To address this question, we analyzed JASPAR database in search of putative transcription factor binding sites within 2 kb region upstream of PRRC2A transcription start site. As a result, transcription factor ATF1 binds to PRRC2A within its promoter region, ≈625 bp upstream to its transcription start site (**Figure** **7**A). The ATFs are a group of bZIP transcription factors that were reported to be involved in tumorigenesis,^[^ ^41^ ^]^ yet the role of ATF1 in CRC has not been fully understood. We performed qRT‐PCR and immunohistochemistry to detect mRNA and protein expression of ATF1, respectively, in normal mouse colons and AOM‐DSS‐induced colon tumors. As expected, ATF1 protein was mostly expressed in the nucleus, and a dramatic increase in both ATF1 mRNA and protein was observed in cancerous tissues (Figure 7B,C). We then confirmed these observations in cancer tissues from CRC patients. Immunostaining showed consistent upregulation of ATF1 in the tissues of CRC patients (Figure 7D). Consistent with *PRRC2A*, analysis of TCGA database also showed elevated expression of *ATF1* in CRC compared to normal samples (Figure 7E), and high levels of ATF1 were associated with poor survival in CRC patients (Figure 7F). These results demonstrated that transcription factor ATF1 was aberrantly upregulated in CRC.

To further examine the regulatory role of ATF1 on *PRRC2A* expression, we performed Spearman correlation analysis based on TCGA database and found that the expression levels of these 2 proteins were positively correlated (Figure 7G). Indeed, overexpression of *ATF1* in HCT116 cells exerted a rise in PRRC2A expression (Figure 7H,I). Conversely, the knockdown of *ATF1* in HCT116 cells significantly reduced the expression levels of PRRC2A and CK1ε (Figure 7J,K). To confirm the direct binding of ATF1 to *PRRC2A* promoter, we mutated the putative binding site within *PRRC2A* promoter and measured its transcriptional activity via luciferase reporter assay in HCT116 cells. Interestingly, ATF1 overexpression significantly stimulated *PRRC2A* promoter activity, while mutations at the putative binding sites dramatically attenuated its activity in both vector‐ and ATF1‐overexpressed cells, suggesting that these binding sites were essential for ATF1 binding and PRRC2A upregulation (Figure 7L). Lastly, ChIP‐qPCR showed that ATF1 protein is recruited to the binding site in *PRRC2A* promoter region (Figure 7M). Taken together, these results demonstrated that elevated ATF1 upregulated *PRRC2A* expression in CRC by directly binding its promoter.

**Figure 8:**
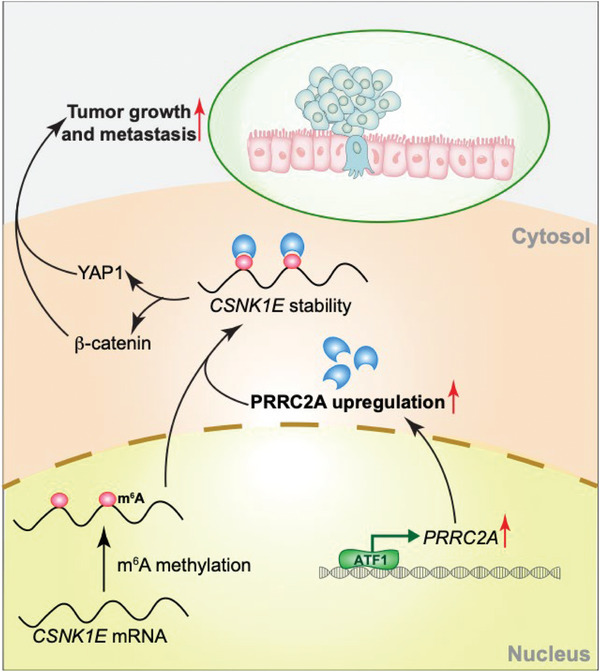
The working model of ATF1‐PRRC2A‐CK1ε axis in driving CRC progression.

In conclusion, we delineated a novel mechanism by which m^6^A reader protein PRRC2A promotes colorectal cancer progression (**Figure** **8**). ATF1 transcriptionally upregulates the expression of PRRC2A, and elevated PRRC2A directly increases the stability of *CSNK1E* mRNA (encoding CK1ε) in an m^6^A‐dependent manner, whereas CK1ε concomitantly activates both WNT and YAP signaling pathways, thus promoting CRC progression. Our study has revealed a previously‐unappreciated mechanism of m^6^A modification in activation of WNT and YAP signaling pathways, as well as in CRC progression.

## 3 Discussion

As the most prevalent internal modification of mammalian mRNA, the reversible m^6^A methylation recently emerged as a novel, fine‐tuning machinery for post‐transcriptional gene regulation.^[^ ^3^, ^42^ ^]^ The orchestration of 3 types of molecules named “writer”, “eraser”, and “reader” allows dynamic regulation of gene expression in adaptation to cellular needs.^[^ ^40^, ^42^ ^]^ Among them, the “readers” are molecules that recognize RNA m^6^A modifications and regulate mRNA stability, splicing, and translation.^[^ ^43^, ^44^, ^45^ ^]^ Increasing evidence indicates that m^6^A modulates oncogenes and tumor suppressor genes in various cancer types.^[^ ^5^, ^42^ ^]^ Notably, global m^6^A levels were increased in gastric and liver cancer cells.^[^ ^46^, ^47^ ^]^ Reader protein such as YTHDF1 was negatively correlated with CD8^+^ T cell infiltration in CRC, and its depletion led to augmentation of anti‐tumor immunity and anti‐PD‐1 efficacy.^[^ ^48^ ^]^ PRRC2A is a newly identified m^6^A reader protein, which is reported to be upregulated in varied cancer types and associated with poor survival.^[^ ^10^ ^]^ However, its roles and mechanisms in cancer progression remain largely unknown. In this work, we found that PRRC2A was upregulated in CRC cells to activate pro‐oncogenic signaling pathways in an m^6^A‐dependent manner, finally contributing to CRC progression. Specifically, we observed that depletion of PRRC2A diminished the expression of cancer stem cell markers and stemness in both chemical and genetic CRC models, as well as in cancer cell lines and organoids. Indeed, a previous report suggested that PRRC2A was critical for oligodendroglial differentiation via directly recognizing m^6^A sites of *Olig2* mRNA and enhancing its stability.^[^ ^9^ ^]^ Likewise, PRRC2A was also suggested as a modulator of spermatogenesis by binding to spermatogonia‐specific transcripts to downregulate their translation.^[^ ^49^ ^]^ Putting together, these observations demonstrated that PRRC2A can serve as a key player in cell stemness maintenance and differentiation in various tissues, by functioning as an m^6^A reader recognizing different downstream targets. Interestingly, we found that intestinal epithelium‐specific deletion of *Prrc2a* had no effect on intestinal stem/progenitor cells in vivo, while *Prrc2a* deletion significantly repressed the organoid‐forming efficiency and growth of cultured murine intestinal and colonic organoids. We speculate that this discrepancy could be due to the compensatory effects of its homologous genes *Prrc2b* and *Prrc2c* in vivo.^[^ ^50^ ^]^ Additionally, the complex microenvironment such as stromal cells could also account for this discrepancy.^[^ ^51^, ^52^ ^]^ It merits further investigation on this point in the future.

Another striking finding is that PRRC2A directly targets CK1ε, which promotes cancer progression by simultaneously increasing WNT and YAP activity. In support of this idea, *CSNK1E* is upregulated in CRC and is involved in advanced progression and poor prognosis.^[^ ^53^, ^54^ ^]^ Importantly, CK1ε is a well‐known positive regulator of WNT signaling pathway that can activate it through multiple mechanisms, including recruiting Dvl‐2 receptor complex,^[^ ^55^, ^56^ ^]^ phosphorylating LRP6,^[^ ^57^ ^]^ and modulating acetylation of β‐catenin.^[^ ^56^ ^]^ Consistent with these observations, inhibiting PRRC2A suppressed WNT activity, while *CSNK1E* overexpression reversed this effect, suggesting that CSNK1E functions as a downstream effector of PRRC2A to downregulate WNT activity.

We also found that elevated PRRC2A promotes YAP1 activity via CK1ε. Specifically, CK1ε can suppress YAP^S127^ phosphorylation, which promotes cytoplasmic retention of YAP1 and activates YAP signaling. Indeed, YAP1 is overexpressed in over 80% of human CRC cases and promotes cancer progression by activating an aberrant core set of enhancers across cancers.^[^ ^22^ ^]^ This idea is also supported by the positive correlation between *CSNK1E* and *PRRC2A* expression in CRC. However, this finding was contradictory to a previous report indicating that CK1δ/ɛ function as negative regulators of YAP1 activity, as phosphorylation of YAP1 at S381 can prime it for phosphorylation by CK1δ/ɛ to activate a phosphodegron, eventually leading to YAP1 degradation, in NIH‐3T3 cells.^[^ ^38^ ^]^ This phenomenon could be due to marked downregulation of the key YAP1 regulators Lats1/2, which are responsible for priming YAP1 by phosphorylating it at S381, in CRC^[^ ^58^ ^]^ and also indicates that CK1ε could have contrasting effects on YAP1 activity depending on the gene network context. Furthermore, it is worth noting that CK1ε is a master‐regulator kinase of the circadian cycle^[^ ^59^ ^]^ and that dysregulation of circadian rhythms increases susceptibility to cancers.^[^ ^60^ ^]^ Thus, we have identified a unique, m^6^A‐dependent mechanism for the simultaneous activation of both canonical WNT and YAP pathways.

## 4 Experimental Section

### Ethics Statement

All mouse experimental procedures and protocols were evaluated and authorized by Beijing Laboratory Animal Management and were performed in strict accordance with the guidelines of the Institutional Animal Care and Use Committee of China Agricultural University (approval number: AW81212202‐3‐1).

### Cell Culture

HCT116 cell lines were purchased from the American Type Culture Collection (ATCC) (Manassas, VA) and cultured in IMDM supplemented with 10% FBS and 1% penicillin‐streptomycin. LoVo cell lines were purchased from the American Type Culture Collection (ATCC) (Manassas, VA) and cultured in DMEM/F12 supplemented with 10% FBS and 1% penicillin‐streptomycin. SW480, HT29, and HEK293T cells were purchased from the American Type Culture Collection (ATCC) (Manassas, VA) and cultured in DMEM supplemented with 10% FBS and 1% penicillin‐streptomycin. NCM460 cell line was purchased from Innovative Life Science Solutions (INCELL) (San Antonio, TX) and cultured in RPMI 1640 medium containing 10% FBS and 1% penicillin‐streptomycin. All cell lines were grown at 37 °C under 5% CO_2_.

### Mice

*Prrc2a* floxed mice were a kind gift from Professor Zengqiang Yuan (The Brain Science Center, Institute of Basic Medical Sciences).^[^ ^31^ ^]^ *Villin‐Cre* mice (stock number: T000142) were purchased from the National Resource Center of Model Mice. *Apc^fl/+^* mice (stock number: 0 08875) were obtained from The Jackson Laboratory. *Vil‐Cre;Prrc2a^fl/fl^* mice were labeled as cKO. Male and female cKO mice were randomly used for all experiments in this study, and the same gender of littermates was used as the control. Mice were housed under SPF conditions with 12 h light/12 h dark cycle, and fed with a regular diet. The housing temperature was 22 °C.

### Plasmid Construction and Cell Transfection

The PRRC2A‐HA vector was synthesized by Shanghai Jierui Biological Engineering Co., Ltd. Prrc2a‐Flag plasmids were a gift from Professor Zengqiang Yuan.^[^ ^31^ ^]^ Flag‐tagged CSNK1E was generated from HEK293T cell cDNA and was cloned and inserted into the pCDNA 3.1 plasmid. To construct the luciferase reporter plasmids, the WT and mutant CSNK1E 3′UTR sequences were inserted into the psiCheck2 luciferase vector. All constructs were verified by DNA sequencing. Short hairpin RNA (shRNA) duplexes designed to target PRRC2A with specific shRNA oligos were synthesized by GenePharma Company (Shanghai, China) and inserted into the pGPH1/GFP/Neo lentiviral vector. All constructs were confirmed by Sanger sequencing.

Cells were transfected with plasmids or siRNAs using Lipofectamine 2000 reagent (Invitrogen) according to the manufacturer's protocol. The media were changed 6 h post‐transfection, and cells were collected after 24 or 36 h.

Sequences used for siRNAs and shRNAs were:

Human shPRRC2A: 5′‐GCTCAAGCTCAGAGCCATTTG‐3′;

Human siPRRC2A‐1: sense 5′‐GCCUGAAAGCCGAGAACAATT‐3′;

Antisense 5′‐UUGUUCUCGGCUUUCAGGCTT‐3′;

Human siPRRC2A‐2: sense 5′‐GCCACCAAUGCGCUUAGUATT‐3′;

Antisense 5′‐UACUAAGCGCAUUGGUGGCTT‐3′;

Human siPRRC2A‐3: sense 5′‐GCUCAAGCUCAGAGCCAUUTT‐3′;

Antisense 5′‐AAUGGCUCUGAGCUUGAGCTT‐3′;

Human siCSNK1E: sense 5′‐CCCGCUACGCUUCCAUCAATT ‐3′;

Antisense 5′‐UUGAUGGAAGCGUAGCGGGTT‐3′;

Mus siPrrc2a: sense 5′‐GGACAAGGCUGCCAAGGAATT‐3′;

Antisense 5′‐UUCCUUGGCAGCCUUGUCCTT‐3′;

Human siMETTL3‐1: sense 5′‐GCAGAACAGGACUCGACUATT‐3′;

Antisense 5′‐UAGUCGAGUCCUGUUCUGTT‐3′.

### Cell Proliferation Assay

The cell proliferation assay was performed using a CCK‐8 Cell Proliferation and Toxicity Assay Kit (Beyotime, C0042). Cells were transfected with shRNAs and/or plasmids for at least 24 h, and 2000 cells were then seeded in 96‐well culture plates and cultured in 100 µL of medium containing 10% FBS. Cell counting was performed at 0, 24, 48, 72, and 96 h, and the absorbance of each well was measured at 450 nm. The absorbance values were proportional to the number of proliferating cells in the culture medium, and cell growth curves were plotted accordingly.

### Spheroid Formation Assay

HCT116 cells (2  ×  10^5^ cells/well) transfected with indicated plasmids or siRNAs were seeded into six‐well ultralow attachment culture dishes (Corning, 3471) and cultured in DMEM/F12 containing 2% B‐27 supplement (Thermo, 17 504 044), 20 ng mL^−1^ EGF (R&D, 236‐EG), 20  ng mL^−1^ basic‐FGF (R&D, 233‐FB) and 1% penicillin‐streptomycin (Gibco, 15 140 122). After 8–11 days, the spheroid colonies were photographed and scored under a microscope (Nikon). Spheroid‐formation efficiency was calculated by the number of spheres that had diameters larger than 75 µm divided by the total cell number in each well.

### In vitro limiting dilution assay

*PRRC2A*‐knockdown or ‐overexpressing HCT116 cells were harvested, and digested into single‐cell suspension, and then seeded at 1, 10, 25, 50, 100, and 250 cells per well into 96‐well ultra‐low attachment plates (Corning, 3474) in 100  µL of serum‐free medium. Fresh medium was added every 4 days. The number of wells with spheroids (diameter ≥ 50 µm) for each group was counted 12 days after plating. The well without spheres (diameter ≥ 50 µm) was defined as a non‐response (*n* = 10). The limiting dilution assays were analyzed using the Extreme Limiting Dilution Analysis (ELDA) online software at http://bioinf.wehi.edu.au/software/elda/.

### Transwell Migration Assay

After transfection with siRNAs and/or plasmids for 24 h, cells were digested with trypsin and then enriched by centrifugation and resuspended in an FBS‐free medium. Cells (2 × 10^5^) were added to Transwell chambers (Corning, 3422), and after 12–24 h, cells that had migrated through the basement membrane were fixed with 4% paraformaldehyde, stained with crystal violet, and photographed for counting.

### APKS Mouse Tumor Organoids Culture and Transfection

The APKS mouse tumor organoids were generated previously by the laboratory.^[^ ^36^ ^]^ For APKS organoid culture, the medium was DMEM/F12 with 1 × B‐27 (Gibco, 17 504 044), 1 × N‐2 (Gibco, 17 502 048), 1 mM N‐acetyl‐cysteine (Sigma‐Aldrich, A9165), 1% Pen Strep (Gibco, 15 140 122), 1 × GlutaMAX (Gibco, 35 050 061), and 10 mM HEPES (Gibco, 15 630 080). The organoids were digested with Accutase enzyme at 37 °C for 8–10 min until they became single cells state. After centrifugation, the supernatant was removed, and cells were resuspended in a 1:1 mixture of pre‐cooled medium and Matrigel (Corning, 356 231) and plated into 48‐well plates. After Matrigel polymerization, 200 µL medium was added to each well. Medium was replaced every 2 days, and organoids were passaged every 4 days.

For APKS organoids transfection, the single cells were transfected using Lipofectamine 2000 reagent (Invitrogen, 11 668 019) with 250 ng Prrc2a‐Flag plasmids or siPrrc2a in one well of a 48‐well plate, following the manufacturer's protocol. After Accutase enzyme digestion, the single cells were incubated with the transfection reagent mixture and reagents in 2 mL centrifuge tubes for 6 h at 37 °C under 5% CO_2_. The supernatant was then removed by centrifugation, and the cells were resuspended in a 1:1 mixture of pre‐cooled medium and Matrigel before being plated into 48‐well plates. After Matrigel polymerization, each well was supplemented with 200 µL medium, and the medium was replaced every 2 days.

### Xenograft Model

Four‐week‐old male BALB/c nude mice were used for subcutaneous xenograft experiments conducted in a specific pathogen‐free (SPF) animal room. The mice were randomly grouped, and HCT116 cells were injected subcutaneously into the right and left dorsal flanks (2 × 10^6^ cells in 100 µL of PBS/mouse). Tumors were harvested 2 weeks after transplantation, and the tumor length, width, and weight were measured. The tumor volume (mm^3^) was calculated as follows: (longest diameter) × (shortest diameter)^2^ × (π/6).

### AOM and DSS Treatment

An AOM–DSS mouse model was generated as described previously with modifications.^[^ ^61^ ^]^ Two‐month‐old Ctrl and *Prrc2a* cKO mice were intraperitoneally injected with AOM (Sigma‐Aldrich) at a concentration of 10 mg k^−1^g body weight. Five days after AOM injection, mice were treated with the so‐called DSS cycle comprising 2 steps: the mice were first given 2% (w/v) DSS (molecular weight: 36000–50000, MP Biomedicals) in drinking water for 7 days and then normal water for 14 days. Mice were subjected to a total of 3 DSS cycles. After treatment, mice were sacrificed to obtain distal colon tissues and tumor numbers and volumes were evaluated.

### Culture for Murine Small Intestinal and Colonic Organoids

Isolation of intestinal and colon crypts was performed as described previously.^[^ ^62^ ^]^ In brief, intestinal crypts were isolated from mice intestine by incubating the tissues in PBS containing 5 mM EDTA for 30 min at 4 °C, and colon crypts were isolated from mice colon by incubating the tissues in PBS containing 5 mM EDTA for 30 min at 37 °C. The crypt fractions were collected through a 70‐µm cell strainer (BD Biosciences). The gathered crypts were washed with PBS and collected by centrifugation at 1000 rpm for 5 min. Then the supernatant was removed. For intestinal crypts, they were resuspended in a 1:1 mixture of ENR medium and Matrigel (Corning, 356 231) and plated into 48‐well plates. For colon crypts, the crypts were resuspended in a 1:1 mixture of WENR medium and Matrigel and plated into 48‐well plates. After Matrigel polymerization, 200 µL of ENR or WENR medium was added to each well. The medium was changed every 2 days, and the organoids were cultured at 37 °C and 5% CO_2_. The ENR medium contained DMEM/F12 supplemented with 50 ng mL^−1^ murine recombinant EGF (PeproTech, 31 509 100), 500 ng mL^−1^ rmR‐spondin1 (R&D, 7 150 250), 100 ng mL^−1^ Noggin (PeproTech, 2 503 820), 10µM Y‐27632 (Sigma‐Aldrich, Y0503), 1 × GlutaMAX (Gibco, 35 050 061), 1 × N2 (Thermo Fisher, 17 502 001), 1 × B‐27 (Gibco, 17 504 044), 10 mM HEPES (Gibco, 15 630 080), 1 mM N‐acetyl‐cysteine (Sigma‐Aldrich, A9165) and 1% Pen Strep (Gibco,15 140 122). The WENR medium was prepared with ENR medium supplemented with Wnt3a (R&D, NP149122).

### RNA Fluorescence In Situ Hybridization

Probes specific to *PRRC2A* were synthesized by ACD. RNA fluorescence in situ hybridization was performed according to the RNAScope Multiplex Fluorescence Assay (ACD, 322360‐USM) following the manufacturer's protocol. In brief, mouse intestinal tissue was embedded in paraffin after fixation with 4% paraformaldehyde for 24 h at 4 °C. Then, the paraffin‐embedded tissues were sliced into 4 µm sections, which were deparaffinized in xylene and an ethanol gradient and were then treated with hydrogen peroxide solution for 10 min at room temperature. Epitope targeting was performed at 100 °C for 15 min prior to protease treatment at 40 °C for 15 min. Probes were hybridized at 40 °C for 2 h, amplified with the RNAScope kit, and detected by Fast Red chromogenic reagent. After dehydration, coverslips were mounted using EcoMount (Biocare, EM897L).

### Histology, Immunohistochemistry and Immunofluorescence Assays

For histological analysis, paraffin‐embedded tissues were sliced into 4 µm sections. These tissue sections were then subjected to hematoxylin and eosin (H&E) staining using standard methods. For immunohistochemical analysis, paraffin sections were deparaffinized in xylene, and an ethanol gradient and antigen retrieval were then performed with an acid repair solution with sodium citrate (pH 6.0) in a microwave oven to expose the surface epitopes. The sections were cooled to room temperature, treated with 3% H_2_O_2_ and 1% Triton X‐100, and blocked with blocking solution (10% normal goat serum in TBS‐T) for 1 h at room temperature. The sections were incubated overnight at 4 °C with the primary antibody. The ABC peroxidase method (Vector Laboratories) was then performed with diaminobenzidine as the enzyme‐substrate and hematoxylin as the counterstain. Finally, the tissue sections were dehydrated and cleared. For immunofluorescence staining, sections were deparaffinized in xylene, and an ethanol gradient and antigen repair was then performed in an acid repair solution with sodium citrate (pH 6.0) in a microwave oven. After cooling to room temperature, the sections were incubated with a blocking solution for 1 h at room temperature. The sections were then incubated overnight at 4 °C in diluted primary antibodies, and the primary antibodies were then detected with Alexa Fluor‐conjugated secondary antibodies. Finally, nuclei were stained with DAPI.

Primary antibodies used for immunostaining include anti‐ATF1 (Abcam, ab181569), anti‐PRRC2A (NOVUS, NBP2‐33471; Abcam, ab188301), anti‐YAP1 (CST, 14 074), anti‐non‐phosphor (Active) β‐catenin (Ser45) (CST, 19 807), anti‐CSNK1E (Abclonal, A1796), anti‐Ki67 (Abcam, ab15580), anti‐Mucin2 (Santa Cruz, sc‐15334), anti‐CD44 (‐1‐APProteintech, 15 675; CST, 37 259), CD133 (Abcam, ab284389), anti‐Cytokeratin 20 (Abcam, ab109111), and anti‐Sox9 (Abcam, ab185966).

### Western Blotting

Cell and tissue lysates were prepared for protein extraction, and protein concentrations were then determined using the BCA method (Beyotime, P0010). Equal amounts of total protein in the samples were separated by 6%–12% SDS‐PAGE and transferred to PVDF membranes (Millipore). The membranes were blocked with 5% skim milk for 1 h at room temperature on a shaker and were then incubated with a primary antibody at 4 °C overnight. After rewarming for half an hour, the membranes were washed 3 times with TBS‐T (10 min each) and incubated with secondary antibody (1:10000, Beyotime) for 1 h. Signals on the membranes were imaged using a LumiQuant AC600 Plus chemiluminescence imaging system and quantified using ImageJ (NIH) software.

Primary antibodies used for Western blotting were anti‐β‐Actin (YEASEN, 30102ES80), anti‐β‐Tubulin (YEASEN, 30301ES80), anti‐PRRC2A (NOVUS, NBP2‐33471; Abcam, ab188301), anti‐YAP1 (CST, 14 074), anti‐p‐YAP1 (CST, 13 008), anti‐non‐phospho (Active) β‐catenin (Ser45) (CST, 19 807), anti‐Lgr5 (Thermo, MA5‐25644), CD133 (Abcam, ab284389), and anti‐CK1ε (Abcam, 270 997).

### RNA Extraction and qRT‐PCR

Total RNA was extracted with TRIzol reagent (CoWin Biosciences, CW0580) prior to trichloromethane extraction, ethanol precipitation, and finally diluted in nuclease‐free ddH_2_O. The nucleic acid concentration and purity were then assessed with a NanoDrop spectrophotometer. RNA was reverse transcribed into cDNA using oligo(dT) primers and M‐MLV reverse transcriptase (Promega, M1705). qRT‐PCR was performed on a LightCycler 96 instrument (Roche, 14 552) using specific primers and SYBR qPCR Mix (Genstar, A311). Relative mRNA levels were determined using the 2^−ΔΔCt^ method and were then normalized to GAPDH mRNA levels. The qRT‐PCR primers are listed in Table S2 (Supporting Information).

### Dual‐Luciferase Reporter Assays

The dual luciferase reporter assay was performed according to the manufacturer's instructions for the Dual‐Glo Luciferase Assay System (Promega, E2920).

For identifying ATF1's binding sites on *PRRC2A* promoter, the sequence for *PRRC2A* was located on Chromosome 6, NC_000006.12 (base pairs 31 620 715.31637771 complement) in the human genome. An ≈2‐kb region upstream of the transcript start site (TSS) was identified as the *PRRC2A* promoter in this study, which was located at Chromosome 6, NC_000006.12 (base pairs 31618715–31620714) and cloned into the pGL3‐Basic reporter constructs. The binding site of ATF1 was located at base pairs 31620083–31620090. The construct was verified by DNA sequencing. Nucleotides at ATF1 binding sites were mutated by carrying out site‐directed mutagenesis. The wild type and mutant plasmids were transfected with phRL‐TK control plasmid to HCT116, respectively. After 36 h, cell lysates were collected to measure the firefly and Renilla luciferase activities. Relative luciferase activity values were calculated by dividing each firefly luciferase activity value by the corresponding Renilla luciferase activity value and then normalizing the result to that in the control group for each assay.

In luciferase assay for regulation of *CSNK1E* by PRRC2A, HCT116 cells were cotransfected with PRRC2A siRNA or overexpression vector, and the CSNK1E 3′UTR plasmid (WT or mutant) in the presence of Lipofectamine 2000. After 36 h, cell lysates were collected to measure the firefly and Renilla luciferase activities. Relative luciferase activity values were calculated by dividing each firefly luciferase activity value by the corresponding Renilla luciferase activity value and then normalizing the result to that in the control group for each assay.

For TOP/FOP flash luciferase assay, HCT116 cells were cotransfected with PRRC2A siRNA or overexpression vector and the TOPflash/FOPflash reporter plasmids in the presence of Lipofectamine 2000. After 36 h of incubation, the luciferase activity was detected using the Dual‐Glo Luciferase Assay System. The results were shown as the ratio of TOP Flash to FOP Flash activity.

For TEAD luciferase assay, HCT116 cells were cotransfected with PRRC2A siRNA or overexpression vector and YAP1‐TEAD plasmid. After 36 h, cells were lysed and subjected to luciferase assay following the manufacturer's instructions (Dual‐Glo Luciferase Assay System). The luciferase activity was normalized to the activity of Renilla luciferase.

### ChIP‐qPCR

Samples for ChIP‐seq were prepared according to the manufacturer's instructions for the SimpleChIP Enzymatic Chromatin IP Kit (CST). In brief, cells were treated with 1% formaldehyde for 10 min at room temperature to crosslink proteins to DNA, and the crosslinking reaction was then quenched by adding glycine to a final concentration of 125 mM. Crosslinked chromatin was then digested into fragments of 150–900 bp with micrococcal nuclease for 20 min at 37 °C with frequent mixing. The sonicated nuclear fractions were divided as input control samples, which were incubated with an anti‐Flag antibody (Abclonal, AE092), an anti‐Histone H3 antibody (as a positive control), and IgG (as a negative control) at 4 °C overnight. The quantity of immunoprecipitated DNA located in the *PRRC2A* promoter region was measured by qRT‐PCR with primers that bind specifically to genomic regions in *PRRC2A* promoter. The primers used for ChIP‐qPCR were:

Forward Primer: 5′‐CGAAGAATGCCTACGCTGCG‐3′;

Reverse Primer: 5′‐TCGCATCCCTTTGCCGTGAG‐3′.

### RIP‐qPCR and RIP‐seq

RIP was performed according to previously published protocols with modifications. The PRRC2A‐HA plasmid was transfected into HEK293T cells at 70% confluence. After 36 h of transfection, the cells were gently washed 3 times with precooled PBS, and 1 mL of lysis buffer (150 mM KCl, 10 mM HEPES (pH 7.6), 2 mM EDTA, 0.5% NP‐40, 0.5 mM DTT, proteinase inhibitor cocktail (1:100), 0.4 U µL^−1^ RNasin) was added. Cells were harvested by scraping with a cell scraper and collected into a 1.5 mL nuclease‐free centrifuge tube. After incubation at 4 °C for 30 min with rotation, the cells were centrifuged at 15 000 × g for 20 min at 4 °C, and the supernatant was removed. Then, 100 µL of the cell lysate (10%) was taken as the input sample and placed on ice. Protein A Dynabeads were then washed twice with 900 µL of NT2 buffer (200 mM NaCl, 50 mM HEPES (pH 7.6), 2 mM EDTA, 0.05% NP‐40, 0.5 mM DTT, 0.4 U µL^−1^ RNasin), resuspended in 100 µL of NT2 buffer, and incubated with 5 µg of an anti‐HA antibody (CST, C29F4) or IgG at room temperature for 1 h. The beads were washed twice with 900 µL of NT2 buffer, and the cell lysate was added to the centrifuge tube containing the beads and incubated at 4 °C for 4 h. Afterward, the beads were washed 5 times with 1 mL of NT2 buffer, and the last wash was conducted by rotating the tube at 4 °C for 5 min. Finally, the input samples and immunoprecipitated beads were resuspended in 150 µL of NT2 buffer with 0.1% SDS and 30 µg of proteinase K and incubated at 55 °C for 30 min. The immunoprecipitated and input RNAs were extracted using TRIzol reagent and precipitated with isopropanol containing glycogen. Finally, the RNAs were subjected to RIP‐qPCR and RIP‐seq analyses.

### MeRIP‐qPCR and MeRIP‐seq

For MeRIP‐seq, AOM‐DSS‐induced colon tumors derived from *Prrc2a* cKO and WT mice were utilized. Total mRNA was extracted with TRIzol reagent (Invitrogen, 15 596 026). The quality and quantity of total RNA were analyzed using a NanoDrop ND‐1000 spectrophotometer. RNA integrity was assessed with a Bioanalyzer 2100, with an RNA integrity number (RIN) of >7.0 considered to indicate acceptable integrity. Poly(A) RNA was purified from 50 µg of total RNA by a two‐step method using Dynabeads Oligo(dT). MeRIP‐seq was performed by LC‐BIO Biotechnology (Hangzhou, China). In brief, the isolated mRNA was cleaved into oligonucleotides of ≈100 nt in length using the Magnesium RNA Fragmentation Module. These RNA fragments were then incubated with an anti‐m^6^A antibody (SYSY, 202 003) in IP buffer (50 mM Tris‐HCl, 750 mM NaCl, 0.5% Igepal CA‐630) at 4 °C for 2 h. The fragments were then incubated with Protein A beads and eluted with elution buffer. The eluted m^6^A‐containing fragments and untreated input pairs of photo segments were transformed into the final cDNA library. Finally, 2 × 150 paired‐end sequencing (PE150) on the Illumina NovaSeq 6000 platform (LC‐Bio Technology CO., Ltd., Hangzhou, China) following the protocol recommended by the vendor was performed. For MeRIP‐qPCR, the enrichment of m^6^A‐containing transcripts was determined by qPCR. The corresponding m^6^A enrichment in each sample was calculated by normalizing to the input.

The primers used for MeRIP‐qPCR were:

Forward Primer: 5′‐ CCACCCACCACCTGCCTG‐3′;

Reverse Primer: 5′‐ CCTGGATTCTGAGGCTCGG‐3′.

### mRNA Stability Assay

Cells were treated with 5 µg mL^−1^ actinomycin D for 0, 3, 6, and 9 h after 24–48 h of transfection. Total RNA was extracted with TRIzol reagent and reverse transcribed into cDNA. qRT‐PCR was used to determine the effect of *PRRC2A* knockdown on the target mRNA degradation rate.

### RNA Pull‐down Assay

The 3′‐biotin‐labelled RNA oligonucleotides containing adenosine or m^6^A (CSNK1E‐ss‐m^6^A: 5′‐ CGGAGAAGUG (m^6^A) CAGGUCCCAG‐3′; CSNK1E‐ss‐A: 5′‐CGGAGAAGUGACAGGUCCCAG‐3′) were synthesized by The Beijing Genomics Institute. The interaction between *CSNK1E* and PRRC2A was detected by Pierce Magnetic RNA‐Protein Pull‐Down Kit (Thermo Fisher Scientific, USA) according to the manufacturer's protocols. Briefly, siNC or siPRRC2A was transfected into HCT116 cells at 70% confluence. After 24 h of transfection, HCT116 cells were harvested, washed with PBS, and lysed in lysis buffer (150 mM KCl, 10 mM HEPES (pH 7.6), 2 mM EDTA, 0.5% NP‐40, 0.5 mM DTT, proteinase inhibitor cocktail (1:100), 0.4 U/µL RNasin). Biotin‐labeled RNA oligonucleotides with/without m^6^A modification were conjugated to streptavidin magnetic beads. RNA‐conjugated streptavidin beads were washed and resuspended in a binding buffer. Afterward, cell lysates were subjected to streptavidin agarose magnetic beads for 1 h at 4 °C with rotation. The beads were eluted by 40 µL 1×SDS buffer at 98 °C for 10 min after 3 washes with 1× wash buffer. Input and RNA‐protein complexes were analyzed by western blotting analysis using the anti‐PRRC2A antibody.

### Statistical Analysis

All experiments were performed at least 3 times. Unless otherwise stated, all data were analyzed, and the values were presented as the means ± standard deviations (SDs). P values were calculated using Microsoft Excel or GraphPad Prism 5. Statistical analysis for all data was analyzed by using paired or unpaired two‐tailed Student's t‐test between 2 groups, or One‐way ANOVA followed by Tukey's test between multiple groups. *p* < 0.05 was considered to indicate a statistically significant difference (*p < 0.05; **p < 0.01; ***p < 0.001). Bioinformatic analyses, namely, GO analysis and KEGG analysis, as well as heatmap generation, were performed using OmicStudio tools (https://www.omicstudio.cn/tool). The potential m^6^A sites were predicted using the online server SRAMP (https://www.cuilab.cn/sramp/).

## Conflict of Interest

The authors declare no conflict of interest.

## Author Contributions

X.W. and S.W. The authors contribute equally. Z.Y. and X.W. performed conceptualization. X.W., Z.Y., M.D. performed methodology. X.W., S.W., Y.P., Z.Y., M.S., X.Y., J.X., S.Z., M.L. and H.Z. performed the investigation. X.W., Z.Y., L.Y. performed visualization. Z.Y., C.L., and L.Y. performed supervision. Z.Y. and X.W. wrote the original draft. Z.Y., X.W., L.Y., M.V.P. Write, reviewed and edited the original draft.

## Supporting information



Supporting Information

Supplementary Table

Supplementary Table

Supplementary Table

Supplementary Table

## Data Availability

Research data are not shared.
