# Supplementary material for: m6A Reader PRRC2A Promotes Colorectal Cancer Progression via CK1ε‐Mediated Activation of WNT and YAP Signaling Pathways
Source: Adv Sci (Weinh). 2024 Nov 24;12(3):2406935. doi: 10.1002/advs.202406935 (PMC11744581; doi:10.1002/advs.202406935)
Supplement: Supplementary file 1 — Supporting Information [file ADVS-12-2406935-s003.docx]

**Supplementary Materials**

Supplementary Materials for

**m^6^A Reader PRRC2A Promotes Colorectal Cancer Progression**

**via CK1ε-mediated Activation of WNT and YAP Signaling Pathways**

Xi Wu *et al.*

*Corresponding author. Email: [zyu@cau.edu.cn](mailto:zyu@cau.edu.cn) (ZY); [yulu@cau.edu.cn](mailto:yulu@cau.edu.cn) (LY); [lvc@cau.edu.cn](mailto:lvc@cau.edu.cn) (CL)

Figure S1. *PRRC2A* overexpression promotes proliferation and migration of CRC cells.

A) Box plots of *PRRC2A* expression in normal colorectal tissues and colorectal tumor tissues based on TCGA data. A total of 41 normal tissue samples and 275 CRC samples were included. ***p < 0.001. B) *In situ* hybridization for *Prrc2a* in AOM-DSS-induced tumors. The dashed line indicates the border between colon tumor tissues (T) and peritumoral tissues (P). The dashed line indicates the border between colon tumor tissues and peritumoral tissues. Scale bar: 300 μm. C-E) The expression of *PRRC2A* in COAD and READ patients with mutations of *TP53* (C), *KRAS* (D), *BRAF* (E) or wild-type status for *TP53*/*KRAS*/*BRAF*. F) Western blotting analysis of PRRC2A in normal colon epithelial cell line NCM460, and CRC lines HCT116, HT29, LoVo and SW480 cells. β-Actin was used as a loading control. G,H) qRT-PCR analysis (G) and Western blotting analysis (H) of *PRRC2A* in HCT116 cells 24 h after *PRRC2A* siRNA treatment. The sequences of the *PRRC2A* siRNAs are listed in Table S2. n = 3. **p < 0.01, ***p < 0.001. I) The motility of LoVo cells transfected with siNC and siPRRC2A were analyzed via scratch wound-healing assay. n = 4. Scale bar: 500 μm. **p < 0.01. J,K) qRT-PCR analysis (J) and Western blotting analysis (K) of PRRC2A in HCT116 cells transfected with the *PRRC2A* overexpression vector for 24 h. n = 3. ***p < 0.001. L) Growth curve of HCT116 cells after transfection of *PRRC2A* overexpression vector. n = 4. ***p < 0.001. M) Transwell assay showing the migration of HCT116 cells transfected with the pCMV-HA and *PRRC2A*-*HA* vectors. N) Quantification of HCT116 cell migration as shown in panel J. n = 5. ***p < 0.001. Scale bar: 200 μm. O) The motility of CT26 cells transfected with the control vector or *Prrc2a* overexpression vector were analyzed via scratch wound-healing assay (left). The wound closure was quantified (right). n = 6. Scale bar: 500 μm. ***p < 0.001. P) Representative images of APKS tumor organoids after *Prrc2a* overexpression at the indicated time points (left). n = 3. Scale bar: 100 μm. Quantification of the organoid area (right). n = 100 organoids at each timepoint. Scale bar: 100 μm. ***p < 0.001. Q) qRT-PCR analysis of *PRRC2A* in xenograft tumors from HCT116 cells treated with shNC or shPRRC2A. n = 3. ***p < 0.001. The data are presented as the means ± SDs. *p < 0.05; **p < 0.01; ***p < 0.001; n.s., p > 0.05. Statistical analysis in panel G was performed by One-way ANOVA followed by Tukey's test; Two-way ANOVA followed by Tukey's test was used in panels L; the rest was done by unpaired Student's t- test.

Figure S2. Deletion of *Prrc2a* in the intestinal epithelium does not influence the proliferation and differentiation of intestinal epithelial cells.

A) Schematic diagram showing the strategy for the generation of intestinal epithelium-specific *Prrc2a* cKO mice. B) qRT-PCR analysis of *Prrc2a* in the intestinal epithelium from control and cKO mice. n = 3. ***p < 0.001. C) qRT-PCR analysis of *Prrc2a* expression in the duodenum, jejunum, ileum, and colon in 8-week-old mice. n = 3. **p < 0.01, ***p < 0.001. n.s., p > 0.05. D) *In situ* hybridization for *Prrc2a* in the duodenum, jejunum, ileum, and colorectum in 8-week-old mice. Scale bar: 30 µm. E) Representative H&E staining of colons in control and cKO mice. Scale bar: 100 μm. F) Quantification of the crypt depth in panel E. n = 51 crypts. n.s., p >0.05. G) Immunostaining for Mucin2 in the colon from control and cKO mice. Scale bar: 100 μm. H) Quantification of the number of Mucin2^+^ cells per crypt in panel E. n = 18 crypts. n.s., p > 0.05. I) Immunostaining for Ki67 in the colon in control and cKO mice. Scale bar: 100 μm. J) Quantification of the number of Ki67^+^ cells per crypt in panel E. n = 85 crypts. n.s., p > 0.05. K) Immunostaining for Sox9 in the colon from control and cKO mice. Scale bar: 100 μm. L) Quantification of the number of Sox9^+^ cells per crypt in panel E. n = 60 crypts. n.s., p > 0.05. The data are presented as the means ± SDs. *p < 0.05; **p < 0.01; ***p < 0.001; n.s., p > 0.05. Statistical analysis in panel C was performed by One-way ANOVA followed by Tukey's test was used in panels C; the rest was done by unpaired Student's t- test.

**Figure S3. Deletion of *Prrc2a* in the intestinal epithelium impaired intestinal and colonic organoids growth.**

A) Representative images of intestinal and colonic organoids from Control and cKO mice. Red arrows indicate aborted organoid debris. Quantification of percentage of organoids formation. n = 4. **p < 0.01; ***p < 0.001. B,C) Representative images of intestinal organoids derived from control and cKO mice at indicated timepoints (B). Quantification of the area size of organoids in panel B (C). Scale bar: 50μm. n = 20. ***p < 0.001. D,E) Representative images of colonic organoids derived from control and cKO mice at indicated timepoints (D). Quantification of the area size of organoids in panel D (E). Scale bar: 50μm. n = 20. ***p < 0.001. F,G) Immunostaining staining of Ki67, Sox9 and Mucin2 in intestinal (F) and colonic (G) organoids cultured 3 days after seeding. Quantification of percentage of Ki67^+^, Sox9^+^ and Mucin2^+^ cells. n = 7. **p < 0.01; ***p < 0.001. The data are presented as the means ± SDs. *p < 0.05; **p < 0.01; ***p < 0.001; n.s., p > 0.05.

**Figure S4. Functional enrichment analysis of PRRC2A-binding mRNAs.**

A) Western blotting was performed to determine the PRRC2A-HA immunoprecipitation efficiency of HA RIP assay in *PRRC2A*-*HA*-overexpressed HEK293T cells. B) GO analysis of PRRC2A-binding mRNAs in HEK293T cells. The data are presented as the means ± SDs. *p < 0.05; **p < 0.01; ***p < 0.001; n.s., p > 0.05.

Figure S5. PRRC2A overexpression enhances WNT and YAP activity.

A) Immunofluorescence staining of non-p-β-catenin in HCT116 cells transfected with the pCMV-HA or *PRRC2A*-*HA* overexpression vectors. Scale bar: 20 μm. The percentage of nuclear β-catenin+ cells was quantified (right). n = 7. ***p < 0.001. B) Western blotting analysis of non-p-β-catenin and PRRC2A in HCT116 cells transfected with the pCMV-HA (NC) or *PRRC2A*-*HA* overexpression (OE) vectors. β-Actin was used as a loading control. C) Spearman correlation analysis of *PRRC2A* and *CCND1* expression (p < 0.001; R = 0.4229) in human CRC tissues based on the TCGA database. D) High expression levels of *CCND1* were significantly correlated with poor survival in CRC patients. p = 0.026. E) Immunofluorescence staining of YAP1 in HCT116 cells transfected with the pCMV-HA (NC) and *PRRC2A*-*HA* overexpression (OE) vectors. Scale bar: 20 μm. The percentage of nuclear YAP1^+^ cells was quantified (right). n = 4. ***p < 0.001. F) Western blotting analysis of p-YAP1^S127^ and YAP1 in HCT116 cells transfected with the pCMV-HA (NC) and *PRRC2A*-*HA* overexpression (OE)vectors. β-Actin was used as a loading control. G) TEAD4 luciferase reporter assay showing that *PRRC2A* overexpression activates YAP1-mediated transcription. HCT116 cells were transfected with the pCMV-HA (NC) and *PRRC2A*-*HA* overexpression (OE)vectors, and co-transfected with *YAP1* constructs for 36 h before harvesting for the luciferase assay. n = 3. ***p < 0.001. H) Immunofluorescence staining of Yap1 and β-catenin in APKS colon tumor organoids. Scale bar: 200 μm. I) Western blotting analysis of p-YAP1 and YAP1 in APKS organoids transfected with siNC and si*Prrc2a*. β-Tubulin was used as a loading control. Histograms showing the p-YAP1/YAP1 ratio. J) qRT-PCR analysis of Hippo-related genes in APKS organoids transfected with siNC and si*Prrc2a*. n = 3. *p < 0.05, **p < 0.01. K to M) Spearman correlation analysis of *PRRC2A* and *YAP1* (p < 0.001; R = 0.3516) in panel K, *PRRC2A* and *TEAD1* (p < 0.001; R = 0.5895) in panel L, and *PRRC2A* and *TEAD3* (p < 0.001; R = 0.4229) in panel M in human CRC tissues based on the TCGA database. N-P) High expression levels of *YAP1* (N, p = 0.00011), *TEAD1* (O, p = 0.00015) and *TEAD3* (P, p = 0.00046) were significantly correlated with poor survival in CRC patients. The data are presented as the means ± SDs. *p < 0.05; **p < 0.01; ***p < 0.001; n.s., p > 0.05.

Figure S6. CK1ε regulates WNT and YAP signaling in CRC.

A) Analysis of TCGA database showing the upregulation of *CSNK1E* in colon cancer at different stages. There were 41 normal tissues and 45 CRC samples in stage1, 110 in stage 2, 80 in stage 3, and 39 in stage 4. ***p < 0.001. B,C) Western blotting analysis of CK1ε, non-p-β-catenin, p-YAP1 and YAP1 in HT29cells transfected with siNC and si*CSNK1E* (B) and in LoVo cells transfected with pCDNA3.1 and *CSNK1E* overexpression vector (C). D,E) qRT-PCR analysis of WNT and Hippo-related target genes in HT29 cells in HT29cells transfected with siNC and si*CSNK1E* (D) or in LoVo cells transfected with pCDNA3.1 and *CSNK1E* overexpression vector (E). n = 3. *p < 0.05; **p < 0.01; ***p < 0.001; n.s., p > 0.05. F) Western blotting analysis of CK1ε and PRRC2A in LoVo cells transfected with NC and *PRRC2A* overexpression vector (OE). G) m^6^A methylation sites in *CSNK1E* were predicted using the SRAMP server. H) Luciferase activity in lysates of WT and *PRRC2A*-overexpressing HCT116 cells transfected with luciferase reporter vectors containing the WT or mutated (MUT) *CSNK1E* 3’UTR sequence. Luciferase activities were measured 36 h after transfection of the indicated constructs. n = 4. ***p < 0.001; n.s., p > 0.05. The data are presented as the means ± SDs. *p < 0.05; **p < 0.01; ***p < 0.001; n.s., p > 0.05. Statistical analysis in panel C was performed by Two-way ANOVA followed by Tukey's test was used in panels H; the rest was done by unpaired Student's t- test.

Other Supplementary Materials for this manuscript includes the following:

Table S1. Patients' information of the CRC tissue microarray.

Table S2. Primers used in this study.

Table S3. PRRC2A-bound RNA transcripts identified in RIP-seq.

**Table S4. Potential PRRC2A target genes list.**

**Raw data**
